# Spermine suppresses *Salmonella*-induced macrophage innate immune responses via inhibition of the cGAS-STING and TLR4 pathways

**DOI:** 10.1128/mbio.00846-26

**Published:** 2026-05-29

**Authors:** Xiao Wang, Mohua Liu, Xiaoya Qu, Jing Hou, Shukun Chen, Tianyuan Chang, Jiayue Li, Jinglei Xia, Xihui Shen, Yao Wang, Lei Xu

**Affiliations:** 1 Shaanxi Key Laboratory of Agricultural and Environmental Microbiology, College of Life Sciences, Northwest A&F University 213667, Yangling, Shaanxi, China; University of Pennsylvania Perelman School of Medicine, Philadelphia, Pennsylvania, USA

**Keywords:** polyamines, spermine, innate immune response, macrophages, cGAS-STING pathway, DNA conformational switching, *Salmonella *Typhimurium

## Abstract

Polyamines integrate metabolism with innate immunity; however, their antibacterial roles remain unresolved. Here, we show that spermine suppresses innate immune responses against *Salmonella enterica* serovar Typhimurium infection in macrophages. Exogenous spermine blunted pro-inflammatory responses and increased intracellular bacterial burden *in vitro* and *in vivo*. Pharmacologic depletion or back-conversion of endogenous polyamines enhanced pro-inflammatory responses, revealing an intrinsic brake. Mechanistically, spermine promoted B-to-Z conformational switching of bacterial genomic DNA, which attenuated cGAS-STING activation; chloroquine or cerium chloride modulations validated DNA topology control. Genetic and pharmacologic dissection indicated that either TLR4 or cGAS-STING is sufficient for spermine’s suppression, whereas their combined blockade abolished it. These findings highlight coordinated immune evasion at the metabolism-DNA interface, offering therapeutic targets for infection control.

**IMPORTANCE:**

In our research, we set out to understand how a small, naturally occurring molecule called spermine shapes the first line of defense against *Salmonella*, a common cause of foodborne illness. We discovered that when *Salmonella* infects our immune cells, it cleverly causes the levels of spermine to drop. When we experimentally added spermine back, we found that it hampered the immune response, allowing the bacteria to thrive. Delving deeper, we uncovered a two-pronged strategy used by spermine. It not only dampens a primary alarm pathway that detects bacteria on the cell surface but also employs a more subtle tactic inside the cell. Spermine changes the physical shape of the bacterial DNA, twisting it into a form that our internal surveillance systems, like the cGAS-STING pathway, cannot easily recognize. This disguise allows the bacteria to go undetected. Our findings reveal a fascinating mechanism of immune evasion and suggest that controlling spermine levels could be a promising therapeutic strategy for either boosting our defenses against *Salmonella* infections or calming inflammatory diseases.

## INTRODUCTION

Polyamines are ubiquitous, positively charged aliphatic amines that support nucleic-acid stabilization, ribosome biogenesis, and proliferation; among them, spermine is the most highly charged and functionally prominent member in eukaryotes (1). Polyamine homeostasis is maintained by a conserved biosynthetic-catabolic network in which ornithine decarboxylase (ODC) generates putrescine, aminopropyl transferases (SRM/SMS) produce spermidine and spermine using dcSAM (S-adenosylmethionine), and SAT1 (N1-acetyltransferase)-PAOX (polyamine oxidase)/SMOX (spermine oxidase) mediate turnover to prevent toxic accumulation (2). Beyond housekeeping roles, polyamines have emerged as context-dependent modulators of innate and adaptive immunity, positioning cellular metabolism at the interface of host defense and pathogen strategy (1).

*Salmonella enterica* serovar Typhimurium (*S*. Typhimurium) remains a major global pathogen, leveraging type III secretion systems (T3SS) encoded on *Salmonella* pathogenicity islands (SPI)-1/2 to invade epithelia and persist within phagocytes (3). Host sensing of *Salmonella* engages Toll-like receptor 4 (TLR4) recognition of lipopolysaccharide (LPS) to activate MyD88- and TRIF-dependent cascades culminating in NF-κB and IRF3/7 responses and induction of inflammatory cytokines and type I interferons (4). In parallel, cytosolic DNA sensing via cyclic GMP-AMP synthase (cGAS) triggers STING-TBK1-IRF3 signaling upon the detection of bacterial genomic DNA or stress-released mitochondrial DNA, amplifying antibacterial innate immune responses during *S*. Typhimurium infection (4–6).

Accumulating evidence links polyamine metabolism to innate immune activation and infection outcomes. Spermine promotes anti-inflammatory programs and M2-like macrophage states, whereas polyamine limitation biases toward pro-inflammatory activation (1, 7, 8). Spermidine attenuates systemic and neuro-inflammation in preclinical settings, underscoring disease-context specificity (9, 10). In intestinal inflammation, dietary spermidine enhances barrier integrity and expands anti-inflammatory macrophage subsets via PTPN2 (11). Pathogen-polyamine crosstalk can be protective or detrimental: gut-derived spermidine tempered inflammation and reduced MRSA burden by reprogramming macrophages, whereas polyamine depletion can heighten ROS-mediated bactericidal activity, illustrating a “two-edged sword” in host-pathogen dynamics (12, 13). Polyamine catabolism may also drive pathology; during *Helicobacter pylori* infection, SMOX-derived H_2_O_2_ and reactive aldehydes promote DNA damage and oncogenic signaling (14).

Mechanistically, spermine can attenuate TLR4 signaling by interfering with proximal complex assembly and downstream ubiquitin signaling, limiting IκBα degradation, and NF-κB nuclear translocation and pathways, such as MAPK, are also dampened (15–17). Spermidine additionally constrains TLR4-NF-κB and NLRP3 inflammasome activity and promotes antimicrobial autophagy via eIF5A (eukaryotic initiation factor 5A) hypusination (18–21). Spermine has also been reported to directly inhibit JAK1, broadly dampening cytokine signaling in inflammatory settings, and may influence chromatin accessibility with implications for immune-gene regulation (22, 23). Recent work reveals a complementary axis whereby spermine’s high charge density remodels DNA topology from the canonical right-handed B-form toward left-handed Z-DNA, which has lower compatibility with cGAS’s dimeric interface, thereby limiting STING-dependent interferon induction (24). Conversely, in certain biochemical contexts, polyamines can condense naked DNA and potentiate cGAS activation, highlighting stimulus and ligand-form dependence (25). However, whether polyamines remodel DNA topology in macrophages to modulate cGAS-STING activation during bacterial infection remains largely unresolved.

Bidirectional manipulation between *S*. Typhimurium and host polyamine metabolism is increasingly appreciated. Infection rewires macrophage polyamine flux—upregulating ODC1 and SAT1 while downregulating SMS—thereby depleting intracellular spermine and shifting pools toward spermidine, a pattern also observed with other bacterial stimuli (26–28). Exogenous spermine has been reported to enhance *S*. Typhimurium T3SS function, suggesting that host-derived polyamines could inadvertently support bacterial virulence (29). Despite these advances, the functional consequences of spermine-induced DNA conformational changes during bacterial infections, particularly in the context of *S*. Typhimurium, remain largely unexplored.

In this study, we aimed to clarify the role of spermine in regulating the host innate immune response to *S.* Typhimurium infection, with a particular focus on its effects on the TLR4 and cGAS-STING pathways and the new mechanism of DNA B-Z conformational switching. Our findings reveal that *S*. Typhimurium infection indeed leads to a reduction in host cell spermine levels and that exogenous spermine supplementation potently suppresses innate immune activation. We demonstrate that this suppression occurs through the concurrent inhibition of both the cGAS-STING and TLR4 pathways. Crucially, we provide evidence that spermine-induced alterations in bacterial DNA conformation are a key mechanism underlying the inhibition of cGAS activation. This was further validated using chemical modulators of DNA conformation, cerium chloride (a Z-DNA inducer), and chloroquine (a B-DNA stabilizer). Collectively, our research unveils a novel mechanism by which the host metabolome, specifically the polyamine spermine, integrates structural DNA dynamics with canonical signaling inhibition to fine-tune innate immunity during bacterial infection. These findings not only advance our fundamental understanding of metabolic-immune crosstalk but also identify potential therapeutic targets for combating antibiotic-resistant pathogens.

## RESULTS

### *S.* Typhimurium alters macrophage polyamine metabolism and reduces the intracellular polyamine pool

**Fig 1:**
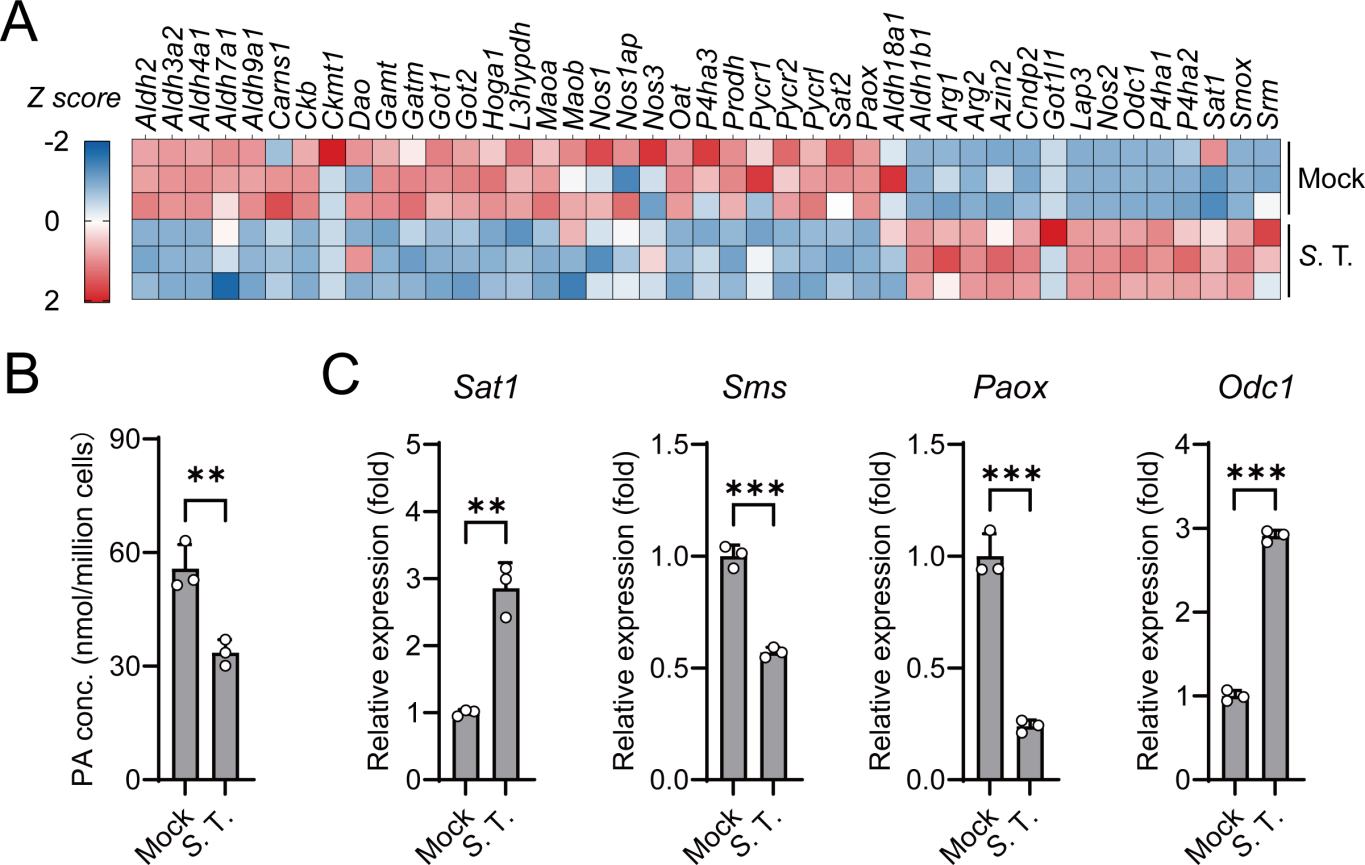
*S*. Typhimurium infection remodels polyamine metabolism and depletes intracellular polyamines in macrophages. (**A**) Heat map showing polyamine-pathway gene expression in PMs infected with *S*. Typhimurium (MOI = 10, 6 h) versus mock. Values are *Z*-scores of relative expressions, with red indicating higher and blue indicating lower abundance. *n* = 3. (**B**) The polyamine levels in PMs infected with *S*. Typhimurium (MOI = 10, 6 h) or mock-infected. *n* = 3. (**C**) The mRNA expression levels of the indicated genes in *S*. Typhimurium-infected (MOI = 10, 6 h) PMs, relative to mock-infected control. *n* = 3. (**B** and **C**) Student’s two-tailed *t*-test. Error bars represent ± SEM. **P < 0.01; ***P < 0.001. See also Fig. S1.

It has been reported that virus-induced reprogramming of polyamine pathways could modulate innate immune response (24). We explored whether the bacterial infection also led to polyamine pathways reprogramming. Using RNA-seq data from our previous study (30) and LPS-stimulated peritoneal macrophages (PMs), we show that both *S*. Typhimurium infection and LPS treatment disrupt host polyamine metabolism (Fig. 1A; Fig. S1A). To explore this further, we quantified intracellular polyamine concentrations in PMs following infection. As illustrated in Fig. 1B, *S.* Typhimurium infection led to a significant reduction in total polyamine levels compared to mock-infected controls. This observation prompted an investigation into the transcriptional mechanisms driving these metabolic alterations. Upon infection, key genes involved in polyamine metabolism displayed marked changes in expression. Specifically, *Sat1*, which encodes spermine/spermidine N1-acetyltransferase 1 (SAT1), the rate-limiting enzyme for spermine and spermidine catabolism, was significantly upregulated (Fig. 1C). In contrast, *Sms*, encoding spermine synthase, responsible for converting spermidine to spermine, and *Paox*, which oxidizes N1-acetylated polyamines, were downregulated, potentially contributing to the declined spermine levels (Fig. 1C). Notably, *Odc1*, encoding ornithine decarboxylase 1 (ODC1), the rate-limiting enzyme in *de novo* polyamine biosynthesis, was also upregulated, indicating a compensatory increase in overall polyamine production that may favor spermidine accumulation over spermine, which is consistent with the previous study also showed that *Odc1* is induced upon *S.* Typhimurium infection (Fig. 1C) (26). This pattern of gene expression aligns with prior reports demonstrating that *S.* Typhimurium manipulates host polyamine metabolism to enhance its intracellular survival. Collectively, our data indicate that *S*. Typhimurium infection induces targeted dysregulation of host polyamine metabolism genes, resulting in reduced spermine levels.

### Exogenous spermine dampens innate cytokine responses and increases *Salmonella* burdens *in vivo*

**Fig 2:**
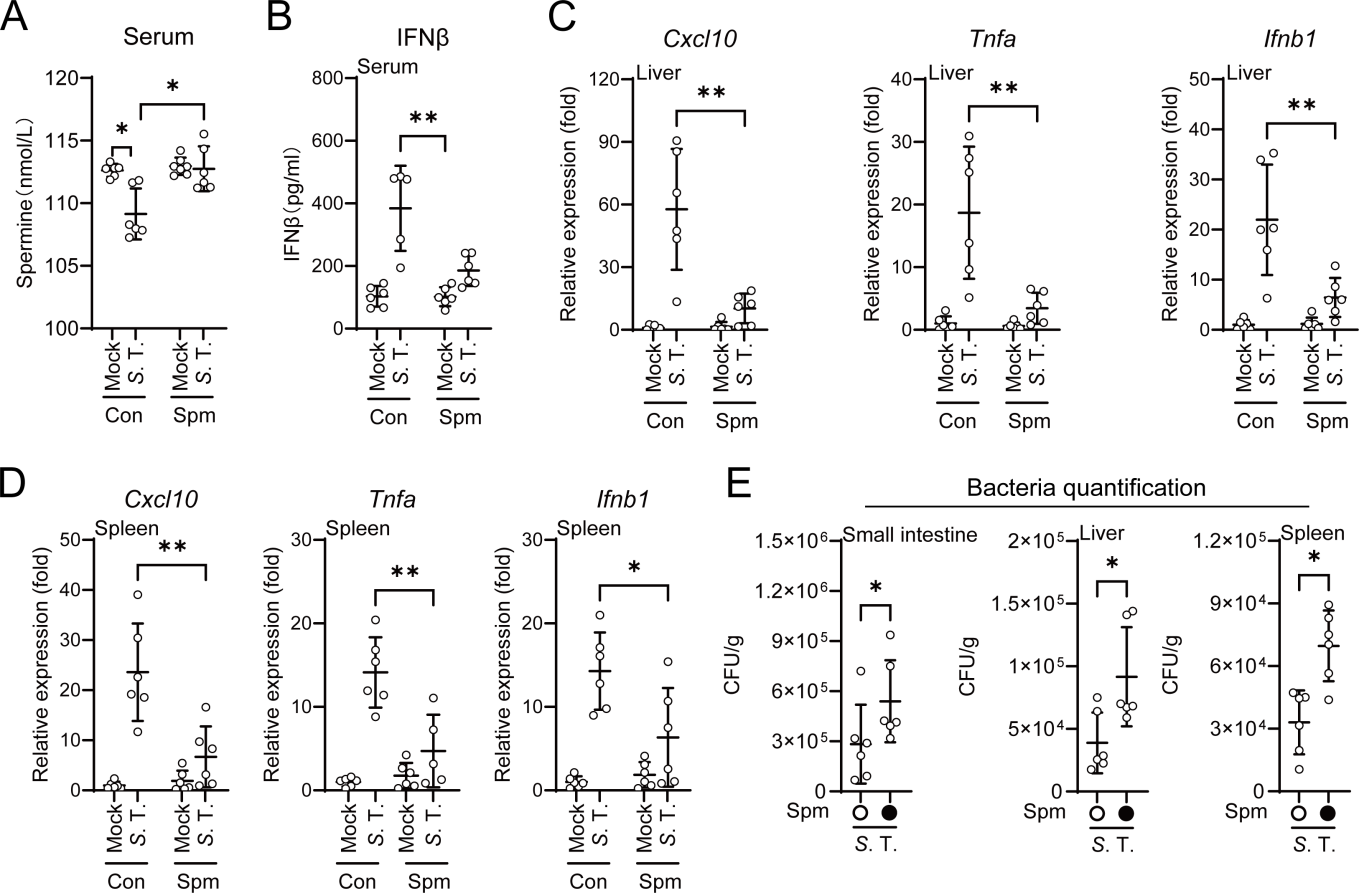
Spermine suppresses infection-induced cytokine responses and increases *Salmonella* tissue burdens. (**A**) The spermine levels in the serum of C57BL/6 mice and spermine-treated mice intragastrically infected with *S*. Typhimurium (2 × 10^9^ CFU, 72 h). *n* = 6. (**B**) The interferon-β (IFN-β) levels in the serum of C57BL/6 mice and spermine-treated mice intragastrically infected with *S*. Typhimurium (2 × 10^9^ CFU, 72 h). *n* = 5 and 6. (**C** and **D**) The mRNA expression levels of the indicated genes in the liver (**C**) and spleen (**D**) from C57BL/6 mice and spermine-treated mice intragastrically infected with *S*. Typhimurium (2 × 10^9^ CFU, 72 h). *n* = 5 and 6. (**E**) The CFU of *S*. Typhimurium in different tissues of C57BL/6 mice and spermine-treated mice intragastrically infected with *S*. Typhimurium (2 × 10^9^ CFU, 72 h). *n* = 5-6. (**A–E**), Mann-Whitney test. Error bars represent ± SEM. *P < 0.05; **P < 0.01. See also Fig. S2.

Given spermine’s reported immunomodulatory activity in antiviral settings (24), we asked whether elevating systemic spermine would similarly tune antibacterial defenses *in vivo*. To this aim, we administered spermine to mice prior to *S*. Typhimurium infection and evaluated its impact on polyamine levels, immune responses, and bacterial burden. HPLC analysis revealed that *S*. Typhimurium infection reduced serum spermine levels, while exogenous administration of spermine effectively restored these levels to near-physiological concentrations (Fig. 2A; Fig. S2A), suggesting that our treatment regimen counteracts the infection-induced depletion of this polyamine. These *in vivo* findings recapitulate the infection-triggered depletion observed in our *in vitro* model using PMs (Fig. 1B).

Given the pivotal role of innate immune responses in controlling bacterial replication, we next examined cytokine production. *S*. Typhimurium infection alone led to diminished IFN-β secretion in mouse sera relative to uninfected controls, consistent with the suppressed responses in PMs (Fig. 2B). Moreover, spermine administration further attenuated infection-induced mRNA expression of key innate immune genes, including *Ifnb1*, *Tnfa*, and *Cxcl10*, in liver and spleen tissues (Fig. 2C and D). Aligning with this immunosuppression, spermine-treated mice exhibited significantly higher bacterial burdens in the small intestine, liver, spleen, and isolated PMs compared to untreated infected controls (Fig. 2E), without directly influencing bacterial growth in culture (Fig. S2B). Together, these results demonstrate that exogenous spermine suppresses antibacterial innate immune responses in a mouse model of *S*. Typhimurium infection, thereby promoting increased bacterial proliferation and host susceptibility.

### Spermine suppresses macrophage inflammatory programs and permits intracellular *Salmonella* outgrowth

**Fig 3:**
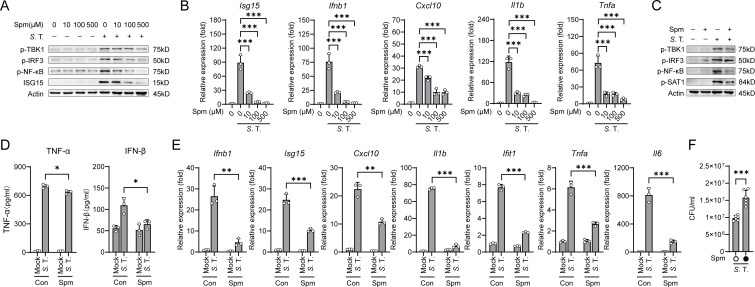
Spermine dampens *Salmonella*-induced innate responses in macrophages and increases intracellular bacterial burden. (**A**) Immunoblot analysis of the indicated antibodies in mouse PMs pretreated with 0, 10, 100, and 500 μM spermine for 2 h and infected with *S*. Typhimurium (MOI = 10, 6 h) or mock-infected. (**B**) The mRNA expression levels of the indicated genes in PMs pretreatment with 0, 10, 100, and 500 μM spermine for 2 h and infected with *S*. Typhimurium (MOI = 10, 6 h), relative to mock-infected control. *n* = 3. (**C**) Immunoblot analysis of indicated antibodies in mouse PMs pretreated with 100 μM spermine for 2 h and infected with *S*. Typhimurium (MOI = 10, 6 h) or mock-infected. (**D**) Enzyme-linked immunosorbent assay (ELISA) analysis of TNF-α and IFN-β production in mouse PMs pretreated with 100 μM spermine for 2 h and infected with *S*. Typhimurium (MOI = 10, 6 h). *n* = 3. (**E**) The mRNA expression levels of the indicated genes in PMs pretreated with 100 μM spermine for 2 h and infected with *S*. Typhimurium (MOI = 10, 6 h), relative to mock-infected control. *n* = 3. (**F**) The CFU of *S*. Typhimurium in PMs pretreated with 100 μM spermine for 2 h and infected with *S*. Typhimurium (MOI = 10, 6 h) or mock-infected. *n* = 3. (**B–F**) Student’s two-tailed *t*-test. Error bars represent ± SEM. *P < 0.05; **P < 0.01; ***P < 0.001. See also Fig. S3.

To investigate the role of spermine in modulating macrophage responses during bacterial infection, we infected mouse PMs with *S*. Typhimurium *in vitro*. Pretreatment with exogenous spermine (Spm) dose-dependently attenuated the bacteria-induced innate immune response. Spermine treatment reduced the phosphorylation of TANK-binding kinase 1 (p-TBK1), interferon regulatory factor 3 (p-IRF3), and nuclear factor kappa B (p-NF-κB), as well as the protein levels of interferon-stimulated gene 15 (ISG15) (Fig. 3A). Consistent with these signaling changes, spermine concentrations as low as 10 μM potently inhibited the mRNA expression of *Isg15, Ifnb1, Cxcl10, Il1b,* and *Tnfa* in a dose-dependent manner (Fig. 3B).

Notably, while suppressing these inflammatory pathways, spermine treatment markedly decreased the phosphorylation of SAT1 (p-SAT1) (Fig. 3C). SAT1 is recognized not only as a rate-limiting enzyme in polyamine catabolism but has also been identified as an interferon-stimulated gene (ISG) (24). At the protein secretion level, ELISA results demonstrated that *S*. Typhimurium infection markedly elevated IFN-β and TNF-α levels in control PMs, but spermine pretreatment significantly diminished this response (Fig. 3D). This suppressive effect was broad, as spermine inhibited the *S*. Typhimurium-induced expression of a panel of type I interferon (*Ifnb1*), ISGs (*Isg15, Cxcl10,* and *Ifit1*), and proinflammatory cytokines (*Tnfa, Il1b,* and *Il6*) in PMs (Fig. 3E).

As expected, this immunosuppressive effect of spermine correlated with enhanced bacterial replication in treated PMs, as evidenced by increased intracellular bacterial loads (Fig. 3F). To determine if this suppression was specific to live bacterial infection or extended to bacterial components, we tested lipopolysaccharide (LPS) stimulation. Spermine pretreatment inhibited LPS-induced expression of ISGs and proinflammatory cytokines in PMs (Fig. S3A). Similar patterns were observed with Enterohemorrhagic *Escherichia coli* (EHEC) EDL933 infection (Fig. S3B). Comparable spermine-mediated suppression occurred in other macrophage models, including RAW264.7 cells (Fig. S3C) and bone marrow-derived macrophages (BMDMs) (Fig. S3D), indicating broad applicability across cell types. Collectively, these results demonstrated spermine as a potent negative regulator of antibacterial innate immune responses in macrophages.

### Pharmacologic reduction of endogenous polyamines relieves the transcriptional suppression of antibacterial responses

**Fig 4:**
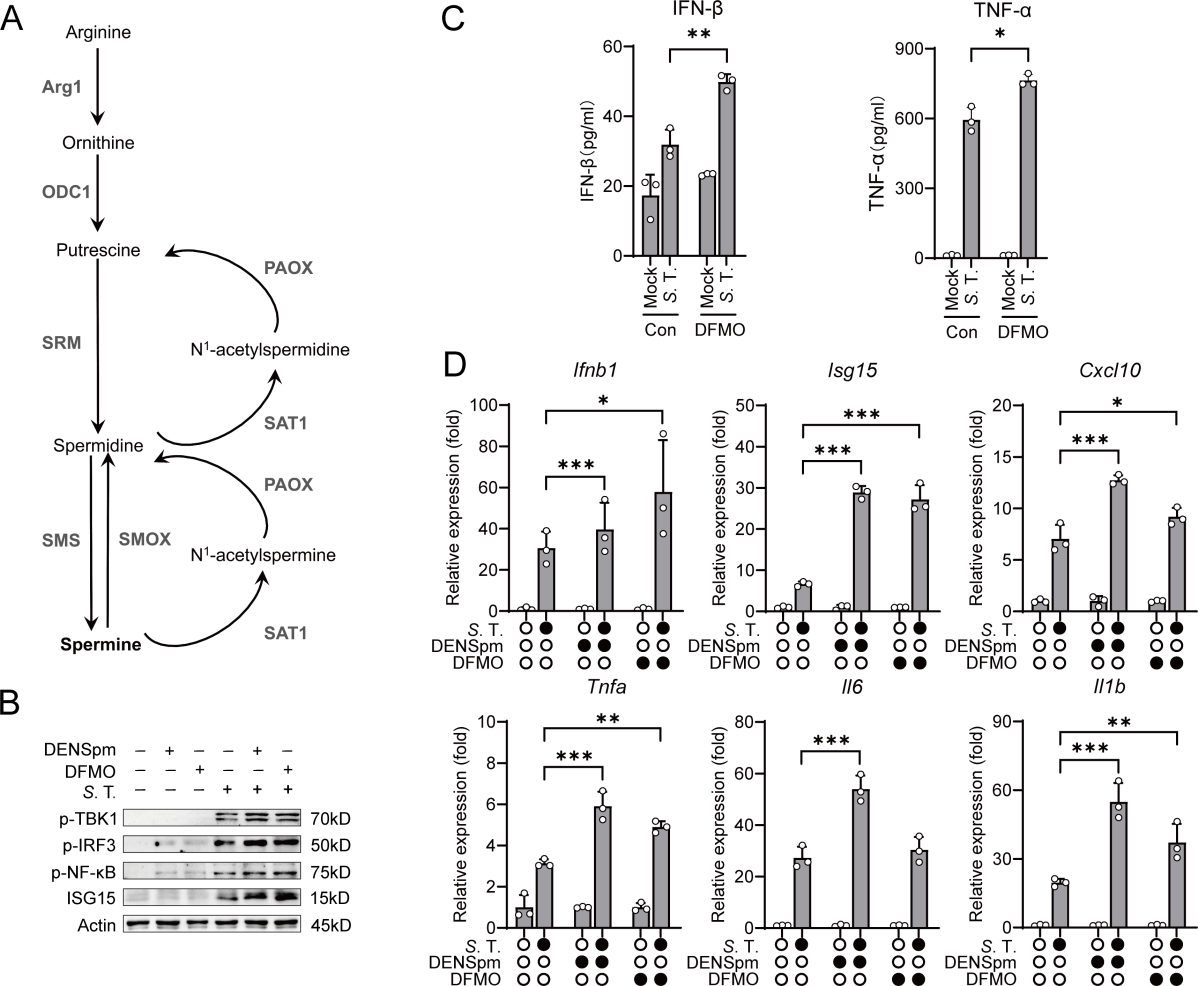
Endogenous polyamines restrain *Salmonella*-induced innate responses in macrophages. (**A**) Schematic of polyamine metabolism. (**B**) Immunoblot analysis of indicated antibodies in mouse PMs pretreated with 10 μM DENSpm for 24 h or 500 μM DFMO for 24 h and infected with *S*. Typhimurium (MOI = 10, 6 h) or mock-infected. (**C**) Enzyme-linked immunosorbent assay (ELISA) analysis of TNF-α and IFN-β production in mouse PMs pretreated with 500 μM DFMO for 24 h and infected with *S*. Typhimurium (MOI = 10, 6 h). *n* = 3. (**D**) The mRNA expression levels of the indicated genes in PMs pretreatment with 10 μM DENSpm for 24 h or 500 μM DFMO for 24 h and infected with *S*. Typhimurium (MOI = 10, 6 h), relative to mock-infected control. *n* = 3. (**C and D**) Student’s two-tailed *t*-test. Error bars represent ± SEM. *P < 0.05; **P < 0.01; ***P < 0.001. See also Fig. S4.

To investigate the role of endogenous polyamines in modulating macrophage immune responses during bacterial infection, we employed two pharmacological inhibitors targeting distinct steps in the polyamine metabolic pathway (Fig. 4A). Difluoromethylornithine (DFMO) inhibits ornithine decarboxylase 1 (ODC1), blocking ornithine-to-putrescine conversion and depleting intracellular polyamines upstream in the biosynthetic pathway. In contrast, N1, N11-diethylnorspermine (DENSpm) activates spermidine/spermine N1-acetyltransferase 1 (SAT1), promoting spermidine and spermine acetylation and back-conversion to putrescine via polyamine oxidase (PAOX) and spermine oxidase (SMOX) (Fig. 4A).

Prior studies have demonstrated that DFMO treatment reduces *S*. Typhimurium colonization and tissue pathology in infected mice while enhancing host survival (26). Consistent with these observations, we found that pharmacologic reduction of polyamines during *S*. Typhimurium infection significantly augmented the innate immune response across multiple regulatory levels. DFMO or DENSpm treatment enhanced the phosphorylation of p-TBK1, p-IRF3, and p-NF-κB and also increased the protein levels of ISG15 compared to infection alone (Fig. 4B). These signaling changes were corroborated at the protein secretion level, as ELISA analysis showed that DFMO treatment significantly elevated the secretion of IFN-β and TNF-α in the culture supernatants (Fig. 4C). Furthermore, both inhibitors markedly upregulated the mRNA expression of a broad panel of innate immune genes, including type I interferon (*Ifnb1*), ISGs (*Isg15* and *Cxcl10*), and proinflammatory cytokines (*Tnfa, Il6,* and *Il1b*) (Fig. 4D). In addition, to determine if these effects extend to bacterial components, we examined LPS-stimulated macrophages. Pretreatment with DFMO or DENSpm similarly augmented LPS-induced expression of proinflammatory cytokines (Fig. S4A). These findings indicate that endogenous polyamines, particularly spermine as the terminal product in the pathway, intrinsically suppress antibacterial innate immune responses in macrophages.

### Spermine-mediated immune suppression during bacterial infection is partially dependent on the cGAS-STING pathway

**Fig 5:**
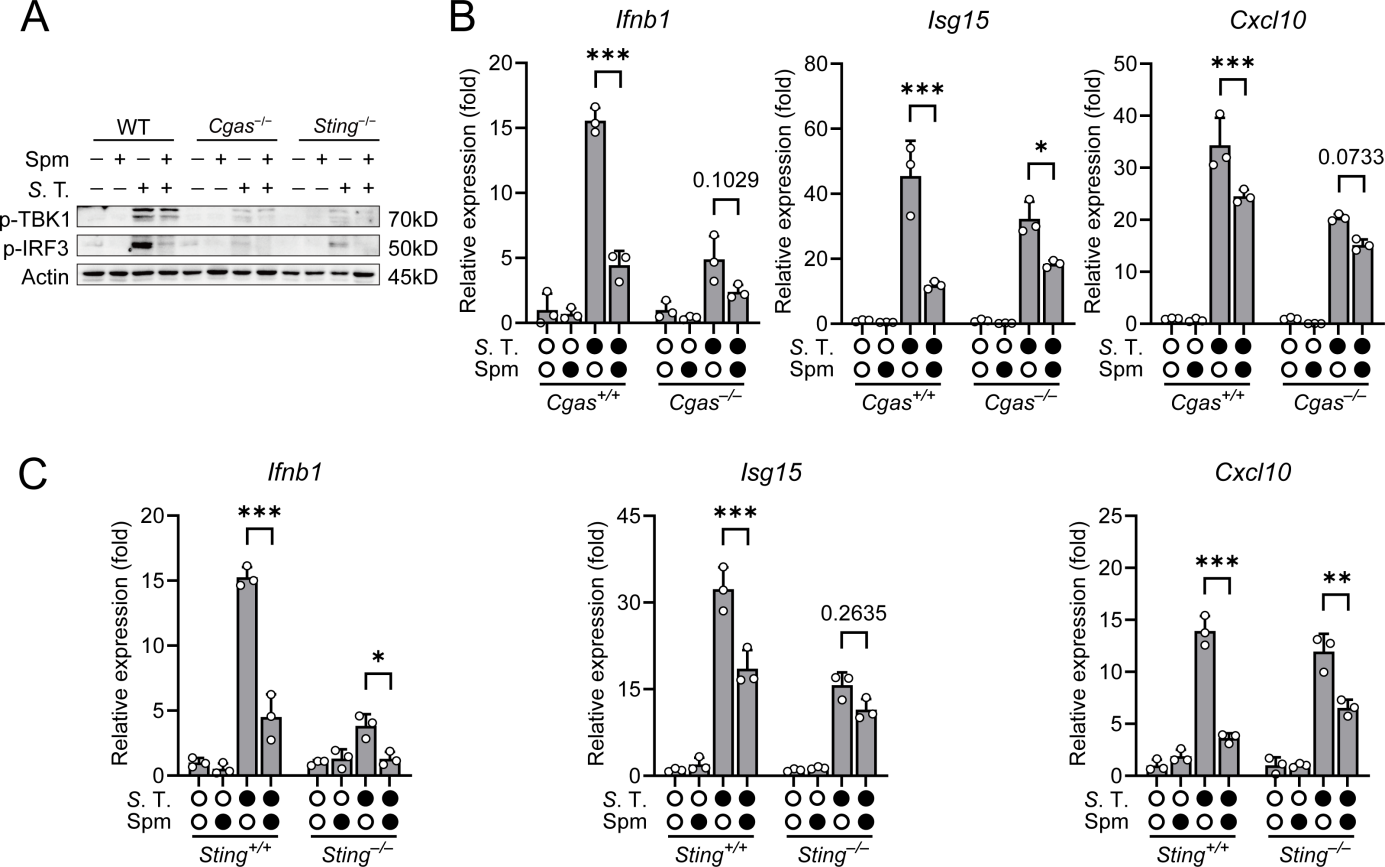
Spermine dampens infection-induced innate responses partly via cGAS-STING. (**A**) Immunoblot analysis of indicated antibodies in *Cgas*^+/+^ or *Cgas*^−/−^ mouse PMs and *Sting*^+/+^ or *Sting*^−/−^ mouse PMs pretreated with 100 μM spermine for 2 h and infected with *S*. Typhimurium (MOI = 10, 6 h) or mock-infected. (**B and C**) The mRNA expression levels of the indicated genes in *Cgas*^+/+^ or *Cgas*^−/−^ mouse PMs (**B**) and *Sting*^+/+^ or *Sting*^−/−^ mouse PMs (**C**) pretreated with 100 μM spermine for 2 h and infected with *S*. Typhimurium (MOI = 10, 6 h), relative to mock-infected control. *n* = 3. (**B** and **C**) Student’s two-tailed *t*-test. Error bars represent ± SEM. *P < 0.05; **P < 0.01; ***P < 0.001. See also Fig. S5.

Previous studies have demonstrated that spermine and spermidine inhibit the production of cyclic GMP-AMP (cGAMP) and selectively attenuate the cGAS-STING signaling pathway (24). Given our earlier observations that *S*. Typhimurium infection activates the cGAS-STING axis in macrophages to drive antibacterial innate immune responses (5), we investigated whether the immunosuppressive effects of spermine during *S*. Typhimurium infection depend on this pathway. To this end, we isolated PMs from wild-type (C57BL/6), *Cgas*^−/−^, and *Sting*^−/−^ mice, pretreated them with spermine, and then infected them with *S*. Typhimurium. Western blot analysis revealed that in WT PMs, spermine pretreatment significantly suppressed the *S*. Typhimurium-induced levels of p-TBK1 and p-IRF3 (Fig. 5A). However, this suppressive effect was markedly attenuated in both *Cgas*^−/−^ and *Sting*^−/−^ PMs. As shown in Fig. 5B, in wild-type (*Cgas*^+/+^) PMs, spermine pretreatment significantly downregulated *S*. Typhimurium-induced expression of type I interferon-associated genes, including *Ifnb1*, *Isg15*, and *Cxcl10*. However, this spermine-mediated suppression was markedly attenuated in *Cgas*^−/−^ PMs, with gene expression levels restored closer to those of *S*. Typhimurium alone, although not entirely abolished. A similar partial rescue was observed in *Sting*^−/−^ PMs (Fig. 5C), where spermine’s inhibitory effects on *Ifnb1*, *Isg15*, and *Cxcl10* were diminished, indicating that the inhibitory effects of spermine are partially mediated through the cGAS-STING pathway. EHEC infection has also been shown to activate innate immune responses via the cGAS-STING pathway, as bacterial DNA sensing by cGAS triggers downstream signaling in infected cells. Consistent with the results for *S*. Typhimurium, the suppressive effects of spermine on EHEC-induced gene expression (*Ifnb1*, *Isg15*, and *Cxcl10*) were partially rescued in *Cgas*^−/−^ and *Sting*^−/−^ PMs, with similar patterns of attenuation in knockout cells (Fig. S5). These results suggest that spermine suppresses host innate immune responses during *S*. Typhimurium infection at least in part by targeting the cGAS-STING signaling axis.

### Spermine promotes Z-DNA conformational changes in bacterial genomic DNA to attenuate cGAS activation

**Fig 6:**
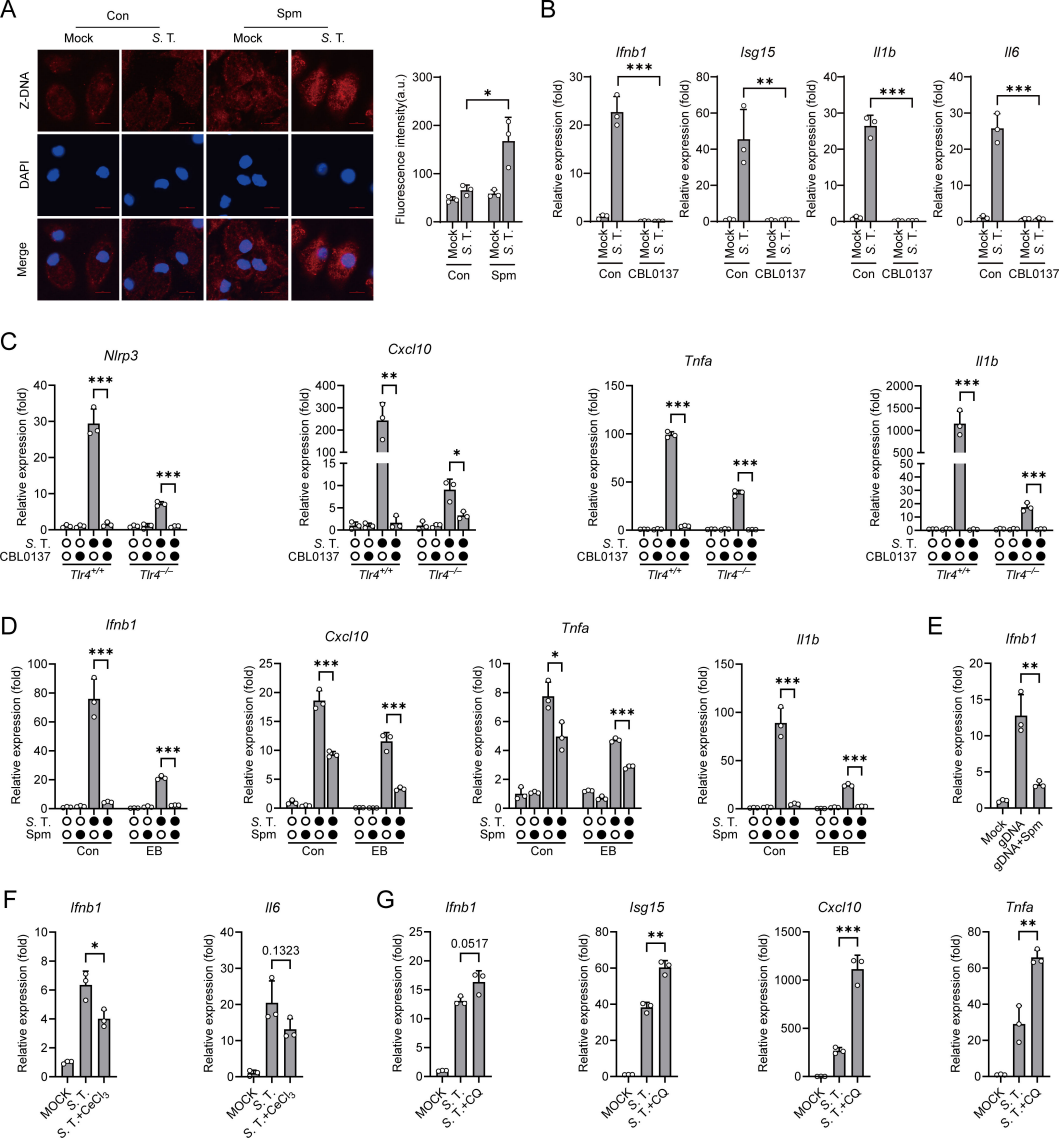
Spermine-induced Z-DNA in bacterial DNA restrains cGAS activation. (**A**) Left: wild-type PMs were treated with 100 μM spermine for 2 h and infected with *S*. Typhimurium (MOI = 10, 6 h) before staining for Z-DNA. Scale bars, 50 μm. Right: quantification of the fluorescence intensity of the Z-DNA signal (left). (**B**) The mRNA expression levels of the indicated genes in PMs pretreatment with 5 μM CBL0137 for 4 h and infected with *S*. Typhimurium (MOI = 10, 6 h), relative to mock-infected control. *n* = 3. (**C**) The mRNA expression levels of the indicated genes in *Tlr4*^+/+^ or *Tlr4^−/−^* mouse PMs pretreatment with 5 μM CBL0137 for 4 h and infected with *S*. Typhimurium (MOI = 10, 6 h), relative to mock-infected control. *n* = 3. (**D**) PMs pretreated with 150 ng/mL ethidium bromide (EB) for 4 days. After changing the new medium, the mRNA expression levels of the indicated genes in PMs pretreated with 100 μM spermine for 2 h and infected with *S*. Typhimurium (MOI = 10, 6 h), relative to mock-infected control. *n* = 3. (**E**) Genomic DNA (gDNA) was incubated with 10 μM spermine, followed by buffer exchange. The mRNA expression levels of the indicated gene in PMs transfected with incubated gDNA (1 μg/mL, 6 h), relative to no-incubated gDNA group. *n* = 3. (**F**) *S*. Typhimurium was cultured in LB with or without CeCl_3_ (500 μM) until the stationary phase. The mRNA expression levels of the indicated genes in PMs pretreated with 100 μM spermine for 2 h and infected with treated or untreated *S*. Typhimurium (MOI = 10, 6 h). *n* = 3. (**G**) *S*. Typhimurium was cultured in LB with or without chloroquine (5 μM) until the stationary phase. The mRNA expression levels of the indicated genes in PMs pretreated with 100 μM spermine for 2 h and infected with treated or untreated *S*. Typhimurium (MOI = 10, 6 h). *n* = 3. (**B–G**) Student’s two-tailed *t*-test. Error bars represent ± SEM. *P < 0.05; **P < 0.01; ***P < 0.001. See also Fig. S6.

To elucidate the mechanisms underlying spermine-mediated suppression of innate immune responses during bacterial infection, we investigated whether spermine induces conformational changes in cytoplasmic DNA that might interfere with cGAS recognition. Given that polyamines can stabilize alternative DNA structures, we specifically focused on the B-to-Z DNA transition, which has been implicated in dampening DNA-induced immune signaling. To rigorously evaluate the presence of Z-DNA, we employed a monoclonal antibody (Z22) specific for the Z-form conformation. As shown in Fig. 6A, *S*. Typhimurium infection triggered a detectable increase in Z-DNA levels within the cytoplasm. Importantly, spermine treatment further significantly enhanced Z-DNA accumulation in both mock and infected cells, demonstrating its potent ability to drive the B-to-Z transition during infection. To further corroborate these findings, we performed spectroscopic analysis by measuring the A295/A260 absorbance ratio of extracted DNA, where an increased ratio serves as an indicator of Z-DNA content. Consistent with our antibody-based results, spermine treatment led to a significant elevation of the A295/A260 ratio (Fig. S6A). Together, these results provide robust evidence that spermine promotes the formation of Z-DNA, which likely reduces the immunogenicity of cytoplasmic DNA and impairs cGAS-mediated innate immune activation.

To mimic and test the functional impact of Z-DNA promotion, we utilized CBL0137, a small molecule that induces Z-DNA formation in mammalian cells while also activating p53 and inhibiting NF-κB. In PMs, CBL0137 treatment suppressed *S*. Typhimurium-induced expression of key ISGs and proinflammatory cytokines, including *Ifnb1*, *Isg15*, *Il6*, and *Il1b* (Fig. 6B). Similar suppression was observed with interferon stimulatory DNA (ISD) stimulation (Fig. S6B), where spermine and CBL0137 reduced *Ifnb1* and *Cxcl10* expression (Fig. S6C). However, CBL0137 also inhibits the gene expression of *Cxcl10* and *Il1b* induced by LPS (Fig. S6D). Since the NF-κB inhibition of CBL0137 could confound results via crosstalk with TLR4 signaling, we validated in *Tlr4*^+/+^ and *Tlr4*^−/−^ PMs. CBL0137 suppressed *S*. Typhimurium-induced *Nlrp3*, *Cxcl10*, *Tnfa*, and *Il1b* in both genotypes (Fig. 6C). These data indicate that Z-DNA promotion contributes to innate immune suppression independently of the TLR4-NF-κB pathways.

Host detection of cytoplasmic DNA, including mitochondrial DNA (mtDNA) and bacterial genomic DNA (gDNA), drives innate responses. Our previous work showed that *S*. Typhimurium infection induces mtDNA release in macrophages, activating nucleic acid sensors to counter pathogen invasion (5). Released mtDNA during bacterial infection can adopt the Z-DNA conformation, and spermine is known to bind and stabilize Z-DNA (31). We hypothesized that spermine might disrupt mtDNA-sensor interactions. To test this, we depleted mtDNA using EB treatment. In EB-treated PMs, spermine retained its suppressive effects on *S*. Typhimurium-induced genes, including *Ifnb1*, *Cxcl10*, *Tnfa*, and *Il1b* (Fig. 6D), indicating mtDNA-independent mechanisms.

This shifted focus to bacterial gDNA as a potential target, given its ability to activate cGAS-STING. Since DNA conformation affects immunogenicity, we transfected PMs with *S*. Typhimurium gDNA pretreated with spermine to induce Z-form (24). Spectroscopic analysis confirmed that spermine treatment of gDNA significantly increased the A295/A260 ratio (Fig. S6E). Crucially, Z-gDNA induced significantly lower *Ifnb1* expression than untreated B-gDNA (Fig. 6E), supporting that Z-DNA is less stimulatory for cGAS. To further confirm, we treated *S*. Typhimurium bacteria with cerium chloride (CeCl_3_) to promote Z-DNA or chloroquine (CQ) to stabilize B-DNA. CeCl_3_ treatment was confirmed to increase the A295/A260 ratio of the bacterial DNA (Fig. S6F). Infection with CeCl_3_-treated bacteria reduced *Ifnb1* and *Il6* (Fig. 6F). Conversely, CQ-treated bacteria enhanced *Ifnb1*, *Isg15*, *Cxcl10*, and *Tnfa* compared to untreated bacteria (Fig. 6G). These results underscore the immunological impact of bacterial DNA conformation. Collectively, these findings demonstrate that during *S*. Typhimurium infection of macrophages, spermine promotes a shift in bacterial genomic DNA from B- to Z-form, thereby inhibiting cGAS-mediated immune activation and suppressing downstream innate responses.

### Spermine-mediated suppression of infection-induced innate responses requires both the TLR4 and cGAS-STING pathways

To elucidate the mechanisms underlying spermine’s immunosuppressive effects during bacterial infection, we noted from prior experiments that while spermine inhibits the cGAS-STING pathway (Fig. 5), it retains partial inhibitory activity on innate immune gene expression in *Cgas*^−/−^ macrophages, suggesting the involvement of additional signaling axes. Previous reports have demonstrated that the related polyamine spermidine ameliorates endotoxin-induced gut barrier dysfunction by suppressing TLR4-mediated proinflammatory signaling (32). This prompted us to investigate whether TLR4 contributes to spermine’s modulation of antibacterial responses.

**Fig 7:**
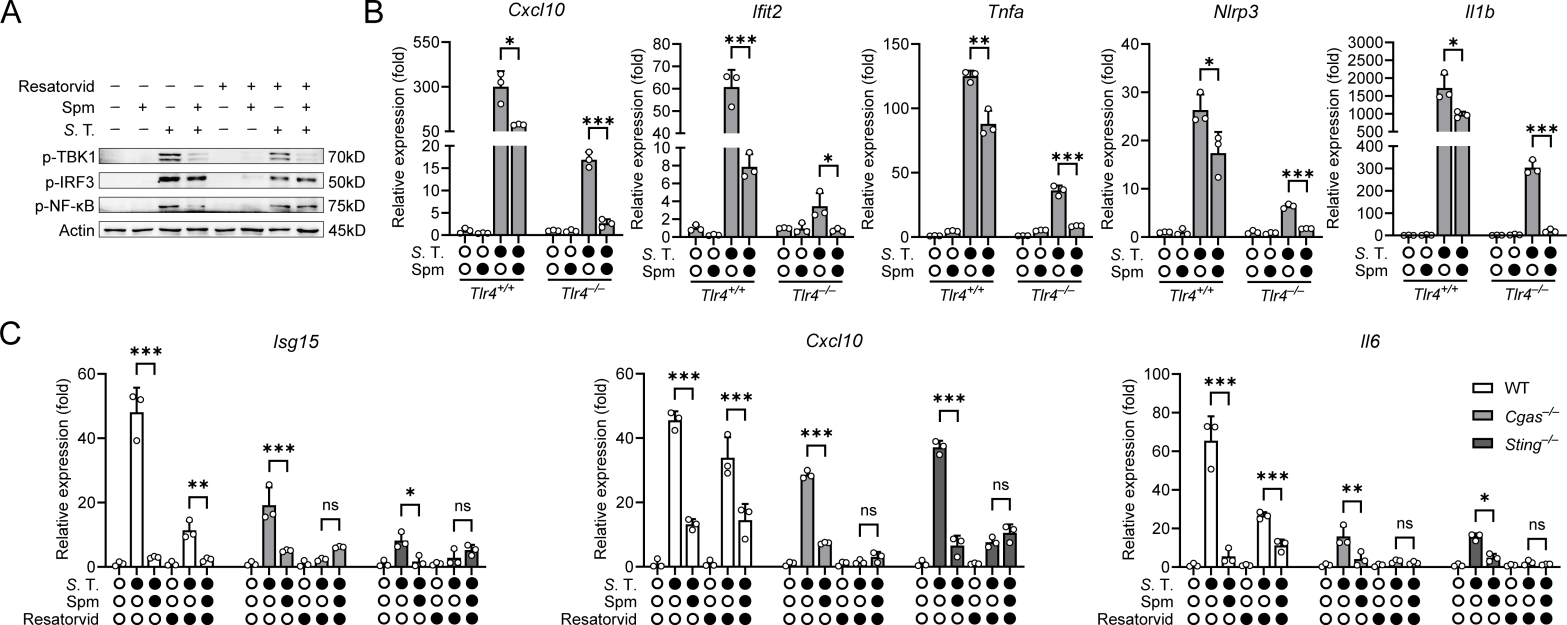
Spermine-mediated suppression requires both TLR4 and cGAS-STING. (**A**) Immunoblot analysis of indicated antibodies in mouse PMs was first treated with 100 nM resatorvid for 12 h, then treated with 100 μM spermine for 2 h, and infected with *S*. Typhimurium (MOI = 10, 6 h) or mock-infected. (**B**) The mRNA expression levels of the indicated genes in *Tlr4*^+/+^ or *Tlr4*^−/−^ mouse PMs pretreated with 100 μM spermine for 2 h and infected with *S*. Typhimurium (MOI = 10, 6 h), relative to mock-infected control. *n* = 3. (**C**) WT PMs, *Cgas^−/−^* PMs, and *Sting^−/−^* mouse PMs were first treated with 100 nM resatorvid for 12 h and then treated with 100 μM spermine for 2 h. The mRNA expression levels of the indicated genes in WT PMs, *Cgas^−/−^* PMs, and *Sting^−/−^* mouse PMs infected with *S*. Typhimurium (MOI = 10, 6 h). *n* = 3. (**B** and **C**) Student’s two-tailed *t*-test. Error bars represent ± SEM. *P < 0.05; **P < 0.01; ***P < 0.001. ns, non-significant. See also Fig. S7.

We first examined the impact of spermine on signaling pathways using western blot analysis. In macrophages infected with *S*. Typhimurium, spermine pretreatment significantly reduced the levels of p-TBK1, p-IRF3, and p-NF-κB (Fig. 7A). Notably, this suppressive effect of spermine was maintained even in the presence of the TLR4-specific inhibitor resatorvid, suggesting that spermine targets signaling molecules downstream of or parallel to multiple receptors (Fig. 7A). To further validate this in a genetic model, we infected peritoneal macrophages from wild-type (*Tlr4*^+/+^) and TLR4-deficient (*Tlr4*^−/−^) mice with *S*. Typhimurium. As shown in Fig. 7B, *S*. Typhimurium infection induced robust expression of ISGs and proinflammatory cytokines/chemokines. Spermine pretreatment significantly suppressed these responses in both *Tlr4*^+/+^ and *Tlr4*^−/−^ macrophages. These results indicate that spermine’s inhibitory effects are not solely dependent on TLR4 signaling.

To test for potential redundancy between pathways, we treated *Cgas*^−/−^ and *Sting*^−/−^ macrophages with the TLR4-specific inhibitor resatorvid during *S*. Typhimurium infection. In WT cells, resatorvid alone had minimal impact on *S*. Typhimurium-induced gene expression, and spermine retained its suppressive effect even with resatorvid (Fig. 7C). However, in *Cgas*^−/−^ macrophages, resatorvid co-treatment largely abolished spermine-mediated inhibition of *Isg15*, *Cxcl10*, and *Il6*. A comparable reversal was observed in *Sting*^−/−^ macrophages, where the addition of resatorvid rendered spermine unable to significantly suppress the infection-induced response (Fig. 7C). These data indicate that the immunosuppressive activity of spermine is mediated by the concurrent modulation of both the cGAS-STING and TLR4 pathways.

To further validate these transcriptional findings at the protein level, we performed ELISA analysis of cytokine secretion in macrophages under various genetic and pharmacological conditions. Consistent with the mRNA data, spermine significantly reduced TNF-α secretion in wild-type cells and those treated with resatorvid alone. However, this suppressive effect was completely abolished in *Sting*^−/−^ macrophages treated with resatorvid (Fig. S7A). Regarding the interferon response, spermine effectively maintained its inhibitory activity on IFN-β secretion even in the presence of resatorvid in wild-type cells (Fig. S7B). However, IFN-β production was virtually undetectable in *Sting^−/−^* macrophages across all treatment groups, underscoring the indispensable role of the cGAS-STING axis in driving the interferon response during *S*. Typhimurium infection.

Extending these findings to another pathogen, we examined EHEC infection in *Tlr4*^+/+^ and *Tlr4*^−/−^ macrophages (Fig. S7C). EHEC induced strong upregulation of *Nlrp3*, *Il6*, *Tnfa*, and *Nos2*, which spermine significantly attenuated in both genotypes, mirroring the TLR4-independent suppression seen with *S*. Typhimurium. Collectively, these data reveal that spermine’s broad immunosuppressive activity during bacterial infection arises from concurrent modulation of both the cGAS-STING and TLR4 pathways.

## DISCUSSION

**Fig 8:**
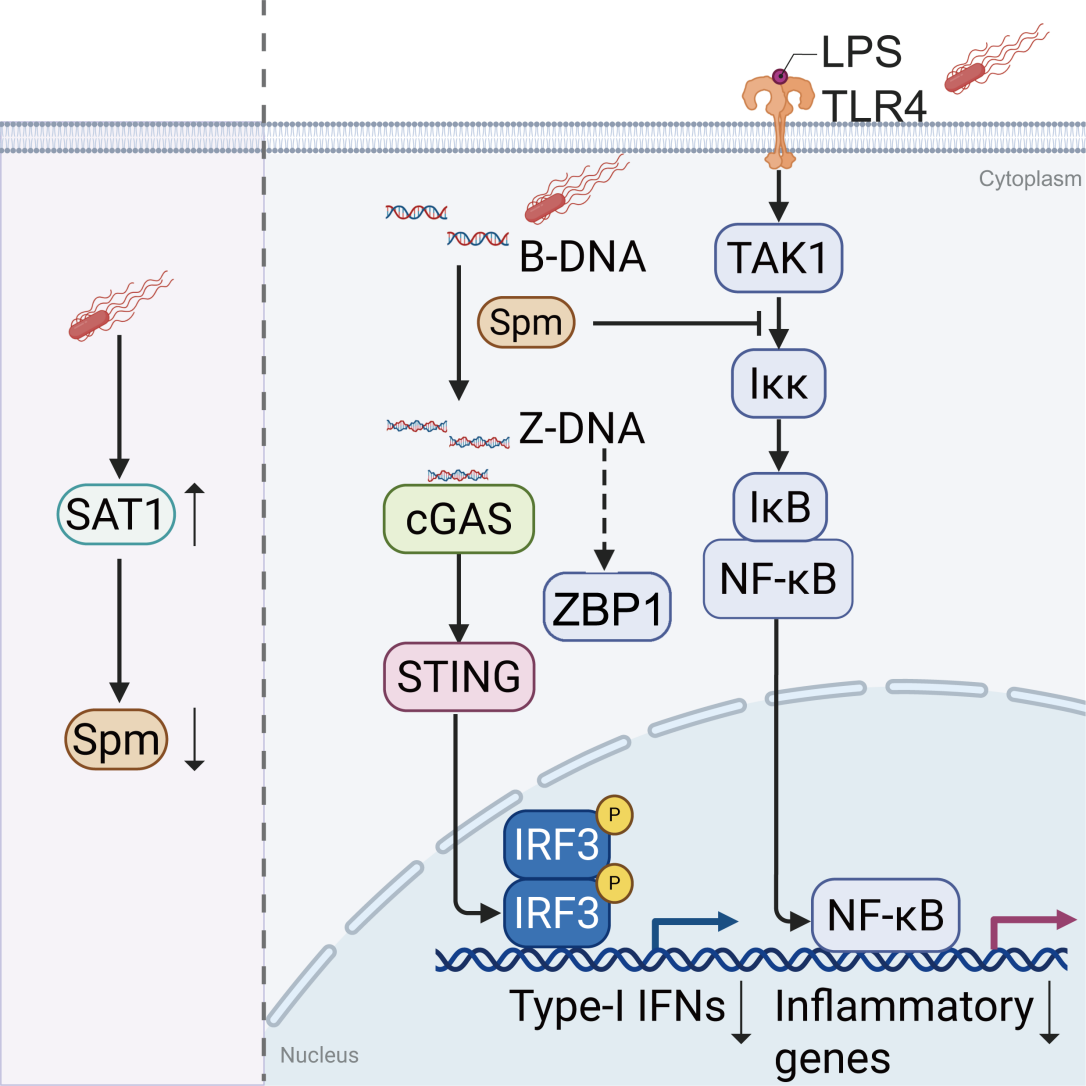
Schematic model of spermine-mediated suppression of macrophage innate immune responses during *S.* Typhimurium infection. *S*. Typhimurium infection reprograms host polyamine metabolism by upregulating *Sat1* (encoding SAT1, the rate-limiting enzyme for spermine catabolism), leading to the depletion of intracellular spermine (Spm) pools. Exogenous or endogenous Spm promotes the conformational transition of bacterial B-DNA to Z-DNA, inhibiting cGAS activation and downstream STING-IRF3 signaling, which reduces type I interferon (IFN) production. The resulting Z-DNA potentially serves as a ligand for alternative sensors such as ZBP1. Concurrently, Spm dampens TLR4-mediated recognition of LPS, NF-κB activation, and inflammatory gene expression. This dual pathway inhibition impairs antibacterial defenses, increasing bacterial burden.

In this study, we demonstrate that spermine acts as a potent modulator of macrophage innate immune responses during *S*. Typhimurium infection, primarily through the concurrent inhibition of the cGAS-STING and TLR4 signaling pathways. Our findings reveal a multifaceted mechanism whereby spermine promotes the conformational transition of bacterial genomic DNA from the immunostimulatory B-form to the less activating Z-form, thereby attenuating cGAS activation and downstream type I interferon (IFN) responses. Additionally, spermine suppresses TLR4-mediated proinflammatory cytokine production, with the full immunosuppressive effect requiring the interplay of both pathways (Fig. 8). These results not only elucidate a novel intersection between polyamine metabolism and nucleic acid sensing but also highlight how *S*. Typhimurium exploits host metabolic reprogramming to dampen antibacterial immunity, potentially facilitating its intracellular survival.

The observed infection-induced reduction in intracellular spermine levels in macrophages aligns with a broader pattern of pathogen-mediated metabolic reprogramming of host polyamine pathways (26, 29). Specifically, *S*. Typhimurium upregulates host genes such as *Sat1* and *Odc1* while downregulating *Sms*, shunting polyamine flux toward spermidine accumulation at the expense of spermine. While other polyamines, such as putrescine and spermidine, are also present in the metabolic pool, our results indicate that their immunomodulatory effects are highly divergent and gene specific, rather than universally immunosuppressive (Fig. S8). This contrast suggests that the metabolic shift from spermine to its precursors may not only represent a loss of a potent immune suppressor but could also involve complex, gene-specific inflammatory regulation. This metabolic shift mirrors strategies employed by other intracellular pathogens, such as *H. pylori*, which induce spermine oxidase (SMOX) to generate reactive oxygen species (ROS) and promote virulence (33). In the context of *S*. Typhimurium, this reprogramming likely serves dual purposes: enhancing bacterial resistance to host ROS through spermidine-mediated antioxidant defenses and reducing spermine availability to limit its immunomodulatory effects (26). Indeed, our data show that exogenous spermine supplementation restores and amplifies immunosuppression, leading to increased bacterial burdens (Fig. 2). This suggests that the pathogen benefits from host-derived polyamines, potentially scavenging them via transporters like PotABCD or PotFGHI, as *S*. Typhimurium lacks endogenous spermine synthase (34, 35). Such bidirectional manipulation underscores the “Janus-faced” role of polyamines in host-pathogen dynamics, where they can either bolster host defenses or be co-opted for microbial evasion (36, 37).

A central mechanism uncovered here is spermine’s ability to induce B-to-Z DNA conformational changes in bacterial genomic DNA, which impairs cGAS binding and subsequent STING activation. This structural modulation, driven by spermine’s high positive charge density interacting with DNA’s phosphate backbone, represents a paradigm shift in understanding metabolic control over innate immune sensing (24). Our experiments with cerium chloride (Z-DNA inducer) and chloroquine (B-DNA stabilizer) confirm that Z-DNA is less immunogenic, leading to reduced expression of ISGs and proinflammatory cytokines during infection. This mechanism extends prior observations in viral contexts, where spermine similarly attenuates cGAS-STING responses to viral DNA (24). However, in bacterial infections, the focus on pathogen-derived genomic DNA rather than mitochondrial DNA (mtDNA) is noteworthy, as mtDNA depletion did not abrogate spermine’s effects (Fig. 6). This specificity may reflect the cytosolic leakage of bacterial DNA during phagosomal rupture, a key event in *S*. Typhimurium pathogenesis (6, 38). Intriguingly, while spermine inhibits cGAS in our model, contrasting studies report its enhancement of cGAS activity by condensing naked pathogenic DNA, suggesting context-dependent effects influenced by DNA source, length, or cellular compartmentalization. Future structural biology approaches, such as cryo-EM, could resolve how spermine-DNA interactions differentially modulate cGAS affinity in bacterial versus viral settings (39, 40)

Given that the B-to-Z transition serves as an “escape” mechanism from cGAS, it simultaneously presents a potential biological trade-off by exposing a different sensing checkpoint involving ZBP1 (Z-DNA binding protein 1). Recent studies have shown that Z-form nucleic acids, such as the Z-RNA generated during Influenza A virus infection, are potent activators of ZBP1-mediated necroptosis via the RIPK3-MLKL axis, a process that must be tightly regulated by enzymes like ADAR1 to prevent inflammatory cell death (41, 42). In the context of *S*. Typhimurium infection, although ZBP1 could potentially sense the spermine-induced Z-DNA, the net outcome observed in our study is a profound suppression of immune signaling and an increase in bacterial burden, rather than massive macrophage necroptosis. This suggests that the attenuation of the cGAS-STING and TLR4 pathways is the dominant effect of spermine during *S*. Typhimurium infection or that the pathogen may further bypass ZBP1-mediated defenses. Future research characterizing the interactions between spermine-induced Z-DNA and various Z-form sensors, including the ZBP1-RIPK3 axis, is necessary to fully define the regulatory role of DNA topology in the innate immune response during bacterial infection.

Complementing the cGAS-STING inhibition, spermine also dampens TLR4 signaling, likely by interfering with adaptor protein recruitment and NF-κB activation, as evidenced by reduced cytokine expression in wild-type but not dual-inhibited cells (20, 43). This dual-pathway targeting is critical, as single knockout of *Cgas* or *Tlr4* only partially relieves suppression, while combined inhibition fully restores immune gene expression. Recent studies have highlighted that polyamines can also influence ISG expression through the downstream JAK-STAT pathway (22). While such downstream modulation likely represents a general metabolic rheostat for cytokine signaling, our findings regarding the B-to-Z transition identify an even earlier, upstream checkpoint specifically targeted at the initiation of DNA sensing. Such crosstalk between TLR4 and cGAS-STING is well-documented in gram-negative infections, where LPS sensing via TLR4 amplifies DNA-mediated IFN responses through IRF3/7 (44, 45). Our results extend this to polyamine regulation, paralleling findings where spermidine (a spermine precursor) blunts TLR4-driven inflammation in macrophages via Nrf2 signaling or autophagy induction (18, 46). Notably, this immunosuppression favors an anti-inflammatory M2 macrophage phenotype, which *S*. Typhimurium may exploit for persistence in granulomas or systemic dissemination (47). These observations are consistent with polyamine roles in other bacterial infections, such as MRSA, where gut-derived spermidine promotes M2 polarization to reduce bacterial burden, highlighting pathogen-specific outcomes.

Our findings suggest potential therapeutic strategies regarding the modulation of polyamine levels. Pharmacological modulation of polyamine levels, such as with DFMO or DENSpm, enhances immune responses and reduces bacterial colonization, supporting their potential as adjunct therapies against antibiotic-resistant *S*. Typhimurium (35, 48). Conversely, exogenous spermine could be harnessed to mitigate hyperinflammation in sepsis or autoimmune conditions driven by excessive cGAS-STING or TLR4 activation (49). However, caution is warranted, as polyamines also influence bacterial virulence; for instance, *S*. Typhimurium utilizes host polyamines to assemble its T3SS, essential for invasion (29, 50). Thus, targeting polyamine transporters or synthases may offer a balanced approach to disrupt pathogen exploitation while boosting host immunity (51).

Despite these advances, limitations persist. Our study primarily utilized murine models and cell lines, necessitating validation in human systems, where polyamine metabolism may differ due to dietary influences or microbiome variations (52, 53). Additionally, while we focused on genomic DNA, the role of other nucleic acids (e.g., RNA) or epigenetic effects of spermine on chromatin accessibility warrants exploration (54). Future research should employ metabolomics and single-cell sequencing to dissect spatiotemporal dynamics of polyamine flux during infection and investigate whether *S*. Typhimurium effectors directly target host polyamine enzymes (55, 56). Moreover, clinical studies correlating polyamine levels with salmonellosis outcomes could inform personalized interventions.

In summary, our work unveils spermine as a key metabolic regulator at the nexus of DNA structural dynamics and innate signaling, providing fresh insights into *S*. Typhimurium immune evasion. By integrating these mechanisms with emerging polyamine-targeted therapies, we may unlock novel strategies to combat bacterial infections and inflammatory disorders.

## MATERIALS AND METHODS

### Mouse studies

All mice were of the C57BL/6 background in this study. Wild-type (WT) C57BL/6 mice were sourced from Beijing Vital River Laboratory Animal Technology Co., Ltd, China. Mice lacking cGAS (*Cgas*^–/–^) and STING (*Sting*^–/–^), also on the C57BL/6 background, were generously provided by Dr. Zhengfan Jiang at Peking University, China. . Mice were housed under standardized conditions (24°C ± 2°C, 12 h light/dark cycle) with unrestricted access to food and water. For the *in vivo* spermine treatment model, 8-week-old female C57BL/6 mice (25–30 g) received intraperitoneal injections of spermine (12 mg/kg) or PBS, administered twice daily over 3 days (22).

### Primary cells and cell lines

Raw 264.7 were cultured in DMEM with 10% FBS, penicillin (100 U/mL), and streptomycin (100 μg/mL). Primary mouse macrophages were isolated following previously established protocols. Bone marrow cells harvested from femurs were cultured for 7 days in DMEM containing 10% FBS, 100 U/mL penicillin, 100 μg/mL streptomycin, 50 μM 2-mercaptoethanol, 2 mM L-glutamine, and 100 ng/mL macrophage colony-stimulating factor (M-CSF) to differentiate into bone marrow-derived macrophages (BMDMs) (30). Reagents used in this study are provided in Table S1.

### Bacterial strains and growth conditions

The bacterial strains employed in this study are detailed in Table S1. The primary strain utilized was *Salmonella* enterica serovar Typhimurium SL1344, which exhibits resistance to streptomycin at a concentration of 100 μg/mL. Additional strains included *E. coli* EDL933, which is susceptible to antibiotics and lacks resistance. All bacterial cultures were grown in Luria-Bertani (LB) broth at 37°C, supplemented with the appropriate antibiotics when necessary. Bacterial proliferation was monitored by measuring the optical density at 600 nm (OD_600_).

### Bacterial infection

Bacterial infection procedures followed previously published methods. *S*. Typhimurium and EHEC were cultured overnight in LB broth at 37°C. For certain experimental conditions, cerium chloride and chloroquine were added to the LB medium at specified concentrations to support bacterial growth. On the following day, the cultures were harvested by centrifugation at 4,500 rpm, washed three times with sterile phosphate-buffered saline (PBS), and subsequently resuspended in either RPMI 1640 or DMEM. At an OD_600_ of 2.0, approximately 10⁹ colony-forming units (CFU) of *S*. Typhimurium are present per milliliter; for EHEC, an OD_600_ of 2.8 yields a similar bacterial count. Prior to infection, PMs and bone marrow-derived macrophages (BMDMs) were washed three times with sterile PBS and maintained in RPMI 1640 medium devoid of fetal bovine serum (FBS) and antibiotics. Cells were then infected with bacteria at specific multiplicities of infection (MOIs) by adjusting the volume of the bacterial suspension accordingly. To synchronize infection, cells were centrifuged at 300 × *g* for 5 min. Following a 30-min incubation at 37°C, the infection medium was replaced with RPMI 1640 containing 10% FBS and gentamicin (200 μg/mL) to eliminate extracellular bacteria. For experiments involving Raw264.7 cells, antibiotic-free medium was used prior to infection. Two hours post-infection, the culture media were exchanged with medium supplemented with 10% FBS and gentamicin (200 μg/mL) for extracellular bacterial clearance.

For intragastric infection in mice, food and water were withheld 8 h before bacterial administration. *S*. Typhimurium cultures were collected, washed twice with sterile PBS, and 2 × 10⁹ CFUs were administered to each mouse via oral gavage using ball-tipped feeding needles. Mice remained fasted until 8 h post-infection, after which access to food and water was restored.

For serum collection, mice were deeply anesthetized using Avertin (tribromoethanol, Sigma-Aldrich T48402, 240 mg/kg, intraperitoneally), following institutional guidelines for anesthesia and analgesia in rodents. Cardiac puncture was performed to obtain whole blood, which was then incubated at 37°C in plastic serum separation tubes for 1 h. After centrifugation at 2,300 × *g* for 5 min, the supernatant (clear serum) was collected and stored at −80°C for downstream analysis.

To quantify bacterial burden in host tissues, mice were euthanized at 72 h post-infection. Organs were excised, weighed, and homogenized in 0.9% sodium chloride solution. Serial dilutions of the homogenates were plated on LB agar containing 200 μg/mL streptomycin to determine CFU counts (5, 30).

### Measurement of intracellular bacterial burden

To measure the intracellular burden of *S*. Typhimurium, macrophages were infected as previously described. The infected cells were lysed using 1% Triton X-100, and the number of bacteria was determined by plating the cell lysates on LB-Miller agar to enumerate viable bacteria (5).

### RNA-seq experiment

Whole transcriptome sequencing was carried out at BGI-Shenzhen (Shenzhen, China). Peritoneal macrophages derived from C57BL/6 mice were infected with *S*. Typhimurium strains at an MOI of 10 for 30 min. For LPS treatment, peritoneal macrophages derived from C57BL/6 mice were transfected with LPS at 100 ng/mL for 6 h. Total RNA was isolated using TRIzol reagent (Sigma-Aldrich, #T9424). RNA quality was evaluated via agarose gel electrophoresis to detect degradation and contamination, while purity and integrity were assessed using a NanoPhotometer and the Agilent Bioanalyzer 2100 system, respectively. The RNA-seq library preparation protocol was based on established procedures. Briefly, mRNA was enriched using oligo(dT)-conjugated magnetic beads, followed by the synthesis of complementary DNA (cDNA), end polishing, addition of adenine at the 3′ ends, and ligation of Illumina-compatible indexed adapters. The resulting PCR products were heat-denatured and circularized to form single-stranded DNA circles (ssCir DNA), which were then amplified using phi29 polymerase to generate DNA nanoballs (DNBs).

These DNBs were loaded onto a patterned nanoarray and sequenced using the BGIseq500 platform to produce 50-base single-end reads. Differential gene expression analysis was conducted using DESeq2, applying a false discovery rate (FDR) threshold of < 0.05 and a minimum fold change of 2. Kyoto Encyclopedia of Genes and Genomes (KEGG) pathway mapping and Gene Ontology (GO) enrichment analyses were performed by BGI using Dr. TOM, a proprietary data analysis platform for transcriptomic data sets.

### RNA isolation and quantitative real-time PCR

Total RNA from mammalian cells was isolated using the RNAeasy Animal RNA Isolation Kit with Spin Column (Beyotime Biotechnology, #R0027). The quality and concentration of RNA were evaluated through agarose gel electrophoresis and quantified using a NanoDrop spectrophotometer (Thermo Scientific). Reverse transcription was carried out with random primers using the Reverse Transcription Kit (Transgene, #AH311-02), and gene expression was measured using the SYBR FAST qPCR Kit (KAPA Biosystems, #KK4601) with gene-specific primers. Quantitative real-time PCR (qRT-PCR) was performed on the Roche LightCycler 96 System. The thermal cycling program consisted of an initial denaturation at 95°C for 150 s, followed by 45 amplification cycles of 94°C for 10 s and 30 s at either 52°C or 58°C, depending on the primer set. For normalization in mammalian cells, Actin served as the internal reference gene. Each sample was analyzed in triplicate, and relative gene expression was calculated using the 2^–ΔΔCt^ method. Primer sequences used in this study are provided in Table S2.

### Western blot analysis

Proteins extracted from the samples were separated via SDS-PAGE and subsequently transferred onto PVDF membranes (Millipore, MA). The membranes were blocked for 2 h at 4°C using QuickBlock Blocking Buffer (Beyotime Biotechnology, #P0252), followed by overnight incubation at 4°C with appropriate primary antibodies. Afterward, the membranes were washed three times with TBST buffer (composed of 150 mM NaCl, 50 mM Tris, and 0.05% Tween-20, pH 7.4) and then incubated with horseradish peroxidase (HRP)-conjugated secondary antibodies for 4 h at 4°C. Protein bands were visualized using enhanced chemiluminescence (ECL) reagents (GE Healthcare, Piscataway, NJ) and imaged using the Tanon 5200 Multi Chemiluminescence Imaging System (Beijing, China).

### Quantification of intracellular polyamine

Polyamine concentrations in macrophages were quantified using a Mouse Polyamine ELISA Kit (Yfxbio Biotech Co. Ltd., #YFXEM00089), following the manufacturer’s recommended protocol. For intracellular polyamine measurement, macrophages were infected with *S*. Typhimurium for 6 h. Cells were then collected, resuspended in PBS to achieve a final concentration of approximately 1 × 10⁶ cells/mL and subjected to repeated freeze-thaw cycles to lyse the cells and release intracellular contents. The resulting lysates were centrifuged for 20 min, and the supernatant was collected for analysis. Absorbance was measured at 450 nm using a kinetic microplate reader (Epoch, Molecular Devices) to determine the polyamine levels.

Serum spermine levels were determined using a modified high-performance liquid chromatography (HPLC) method with pre-column benzoylation, as previously described (57). Briefly, 100 μL of mouse serum was mixed with 100 μL of chilled 5% (vol/vol) perchloric acid to precipitate proteins. The mixture was incubated on ice for 1 h and then centrifuged at 10,000 × *g* for 40 min at 4°C. For derivatization, 200 μL of the resulting supernatant was neutralized with an equal volume of 2 M NaOH, followed by the addition of 5 μL of benzoyl chloride. The mixture was vortexed and incubated at 37°C for 25 min. The derivatized polyamines were extracted using saturated NaCl and diethyl ether. The upper ether phase was collected, evaporated to dryness under a stream of nitrogen, and the residue was reconstituted in 100 μL of methanol. The solution was filtered through a 0.22 μm nylon membrane before analysis. HPLC analysis was performed using a Waters HPLC system (W2690/5 pump, 2998 UV detector) equipped with a SymmetryShield RP18 column (4.6 × 250 mm, 5 μm). The column temperature was maintained at 30°C. The mobile phase consisted of methanol (51%) and ultrapure water (49%) at a flow rate of 0.7 mL/min for a 20-min run. Spermine was detected by UV absorbance at 254 nm. The concentration of spermine was calculated by comparing peak areas with those of known spermine standards processed under the same conditions.

### Measurement of IFN-β by ELISA

IFN-β and TNF-α levels were quantified using a commercial ELISA kit specific for mouse IFN-β (CUSABIO, #CSB-E04945m) and mouse TNF-α (Thermo Fisher Scientific, #88-7324), following the manufacturer’s recommended protocol. For *in vivo* measurements, serum samples were used directly. In *in vitro* assays, culture supernatants from treated cells were collected and subjected to analysis. According to the kit instructions, either serum or cell supernatants were added to ELISA plates pre-coated with capture antibodies, followed by sequential incubation with biotin-conjugated detection antibodies and horseradish peroxidase (HRP) solution, with appropriate wash steps between each stage. Subsequently, the TMB substrate was applied and incubated in the dark to allow color development. The reaction was terminated by adding the stop solution, and absorbance was measured at 450 nm using a microplate reader (Epoch, Molecular Devices) within 5 min of stopping the reaction.

### Incubation and determination of Z-DNA

Genomic DNA (gDNA) from *S*. Typhimurium was extracted using the TIANamp Bacteria DNA Kit (TIANGEN, #DP302), and endotoxin contaminants were removed with the Protein Endotoxin Removal Kit (Beyotime, #C0268S). The induction of Z-form DNA followed the previously described protocols (24, 58), with minor modifications. Specifically, for spermine-mediated induction, gDNA at a concentration of 20 mg/mL was incubated overnight at 37°C with 10 mM spermine in a cacodylate buffer composed of 1.0 mM sodium cacodylate, 50 mM NaCl, and 0.15 mM EDTA (pH 7.4). Following incubation, buffer exchange was performed using Amersham Nap-5 Sephadex columns (#17085301, Cytiva, Marlborough, MA, USA). Quantification of gDNA was carried out with the dsDNA High Sensitivity Assay Kit (Yeasen, #12640ES60). Macrophage transfection was subsequently performed using the EZ Cell Transfection Reagent (Life-iLab, #AC04L091).

To assess intracellular Z-DNA levels post-infection, cells were lysed in 1% Triton X-100, and total DNA was extracted using the TIANamp Bacteria DNA Kit (TIANGEN, #DP302), which does not differentiate between sources. The presence of Z-DNA structures was evaluated by measuring the absorbance ratio at 260 nm to 295 nm, using a NanoDrop One/OneC Microvolume UV-Vis Spectrophotometer, as previously described (59, 60). DNA samples exhibiting a higher 260/295 nm absorbance ratio were indicative of Z-DNA formation, whereas lower ratios were characteristic of B-DNA. Buffer-only samples were used as blank controls.

### Mitochondrial depletion

PMs were cultured in RPMI 1640 medium with or without 150 ng/mL EB for 4 days (61, 62). On Day 4, the cells were washed with PBS and then changed to RPMI 1640 medium without EB. Cells were infected with *S*. Typhimurium on the second day. Total RNA was isolated using the RNAeasy Animal RNA Isolation Kit with Spin Column (Beyotime Biotechnology, #R0027).

### Immunofluorescence staining and confocal microscopy

PMs were seeded onto glass coverslips in 24-well plates and pre-treated with spermine before being infected with *S*. Typhimurium. At 6 h post-infection, the cells were fixed with 4% paraformaldehyde (PFA) for 10 min at room temperature and subsequently permeabilized with 0.5% Triton X-100 in PBS for 15 min. To minimize non-specific binding, the cells were incubated with immunostaining blocking buffer containing saponin (P0104, Beyotime, Shanghai, China) for 1 h. The cells were then incubated with a primary rabbit monoclonal antibody against Z-DNA/Z-RNA (Ab00783-23.0, Absolute Antibody, UK) at 4°C overnight. After three washes with PBS, the cells were incubated with a conjugated goat anti-rabbit IgG (H + L) secondary antibody (ZF-0316, ZSGB-BIO, Beijing, China) for 1 h at 37°C. Finally, the coverslips were mounted using Antifade Mounting Medium with DAPI (P0131, Beyotime) to visualize the nuclei and protect the fluorescence from fading. Images were acquired using a Nikon confocal laser scanning microscope. The fluorescence intensity of cytoplasmic Z-DNA was quantified using ImageJ software (NIH, Bethesda, MD, USA).

### Statistical analysis

Statistical analyses were performed using GraphPad Prism Software (GraphPad Software, San Diego, CA, USA). All experiments were performed in at least three independent replicates. Statistical analyses in mice were performed using the Mann-Whitney test. All other experiments were analyzed using an unpaired, two-tailed Student’s *t*-test. Error bars represent ± SEM. *P < 0.05; **P < 0.01; ***P < 0.001.

## Data Availability

Raw sequencing data have been deposited in the NCBI Sequence Read Archive (SRA) under BioProject IDs PRJNA1047955 and PRJNA1304485.

## SUPPLEMENTAL MATERIAL

Fig. S1 mbio.00846-26-s0001.tif LPS stimulation rewires macrophage polyamine metabolism.

Fig. S2 mbio.00846-26-s0002.tif Spermine does not directly affect bacterial growth.

Fig. S3 mbio.00846-26-s0003.tif Spermine blunts pro-inflammatory response in macrophages.

Fig. S4 mbio.00846-26-s0004.tif Endogenous polyamines restrain LPS-induced innate responses in macrophages.

Fig. S5 mbio.00846-26-s0005.tif Spermine-mediated immune suppression against EHEC infection is partially dependent on the cGAS-STING pathway.

Fig. S6 mbio.00846-26-s0006.tif Spermine and Z-DNA formation attenuate ISD-induced innate immune responses.

Fig. S7 mbio.00846-26-s0007.tif Spermine inhibits EHEC-elicited innate immune response irrespective of TLR4 status.

Fig. S8 mbio.00846-26-s0008.tif Effects of putrescine and spermidine on *S*. Typhimurium-induced gene expression in macrophages.

Fig. S9 mbio.00846-26-s0009.tif Uncropped versions of immunoblotting results.

Supplemental material mbio.00846-26-s0010.docx Legends for Fig. S1-S9; Tables S1 and S2.

ASM does not own the copyrights to Supplemental Material that may be linked to, or accessed through, an article. The authors have granted ASM a non-exclusive, world-wide license to publish the Supplemental Material files. Please contact the corresponding author directly for reuse.
