# Supplementary figures and images for: Spermine suppresses Salmonella-induced macrophage innate immune responses via inhibition of the cGAS-STING and TLR4 pathways
Source: mBio. 2026 May 29;17(7):e00846-26. doi: 10.1128/mbio.00846-26 (PMC13343856; doi:10.1128/mbio.00846-26)

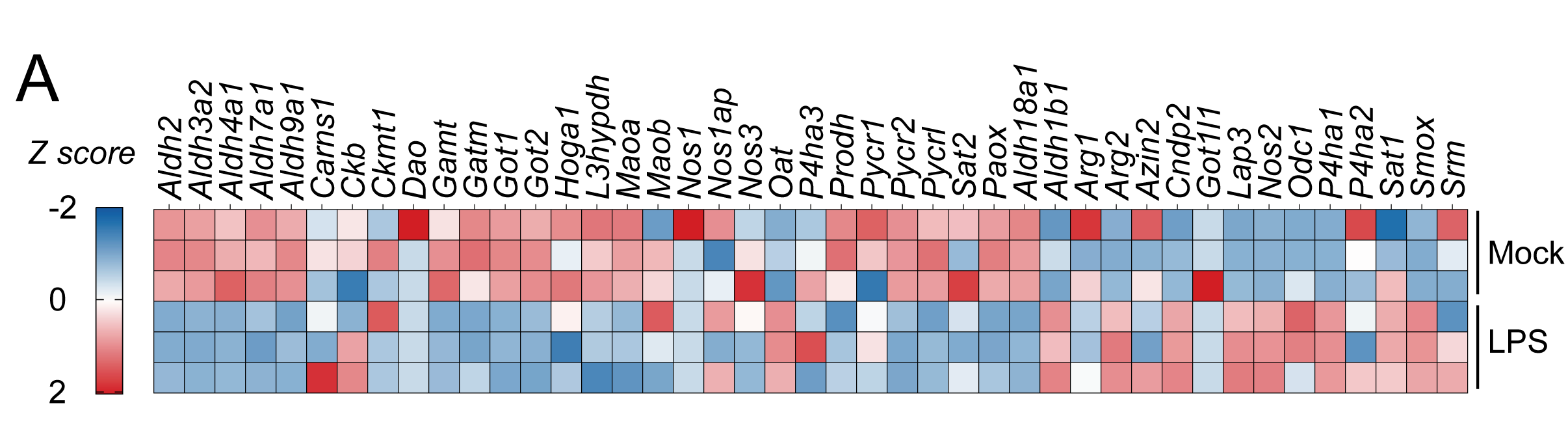

Supplement: Fig. S1 — LPS stimulation rewires macrophage polyamine metabolism. [file mbio.00846-26-s0001.tif]

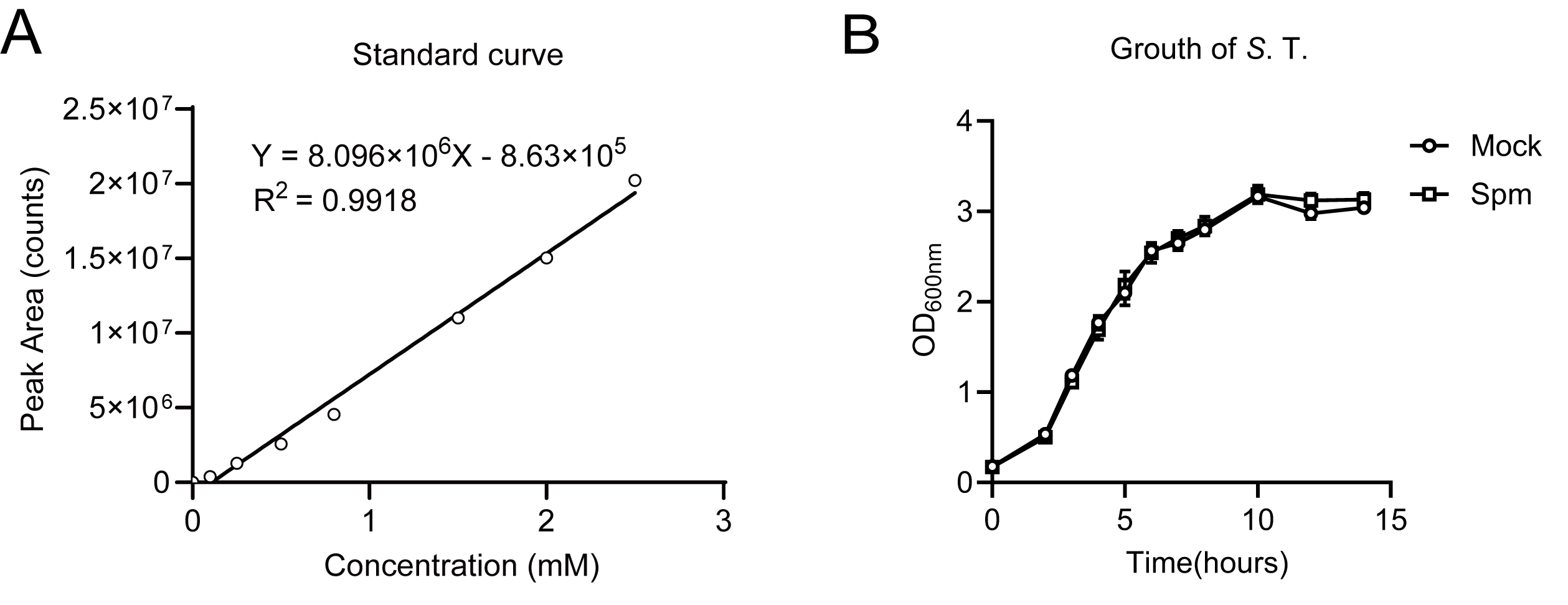

Supplement: Fig. S2 — Spermine does not directly affect bacterial growth. [file mbio.00846-26-s0002.tif]

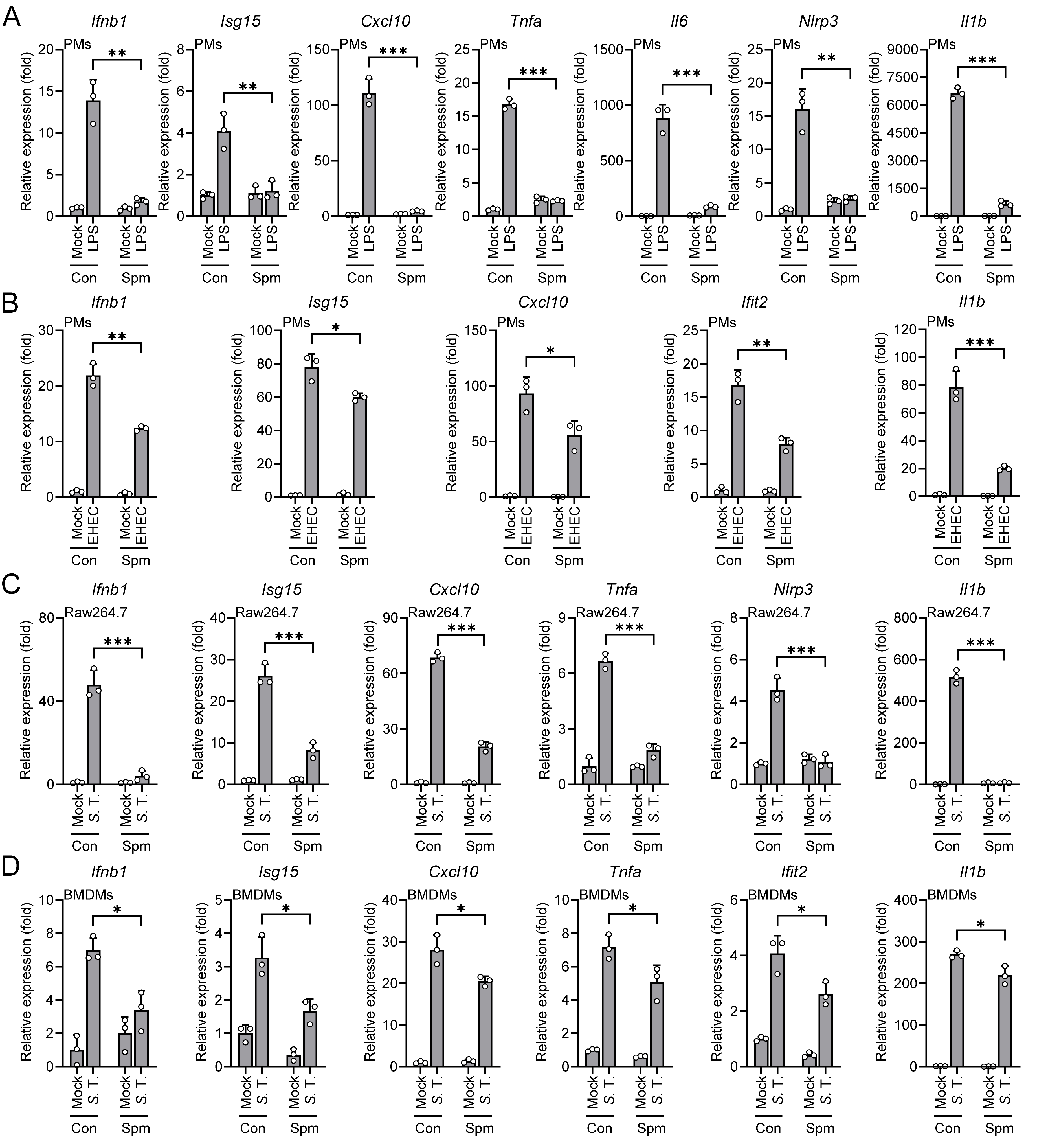

Supplement: Fig. S3 — Spermine blunts pro-inflammatory response in macrophages. [file mbio.00846-26-s0003.tif]

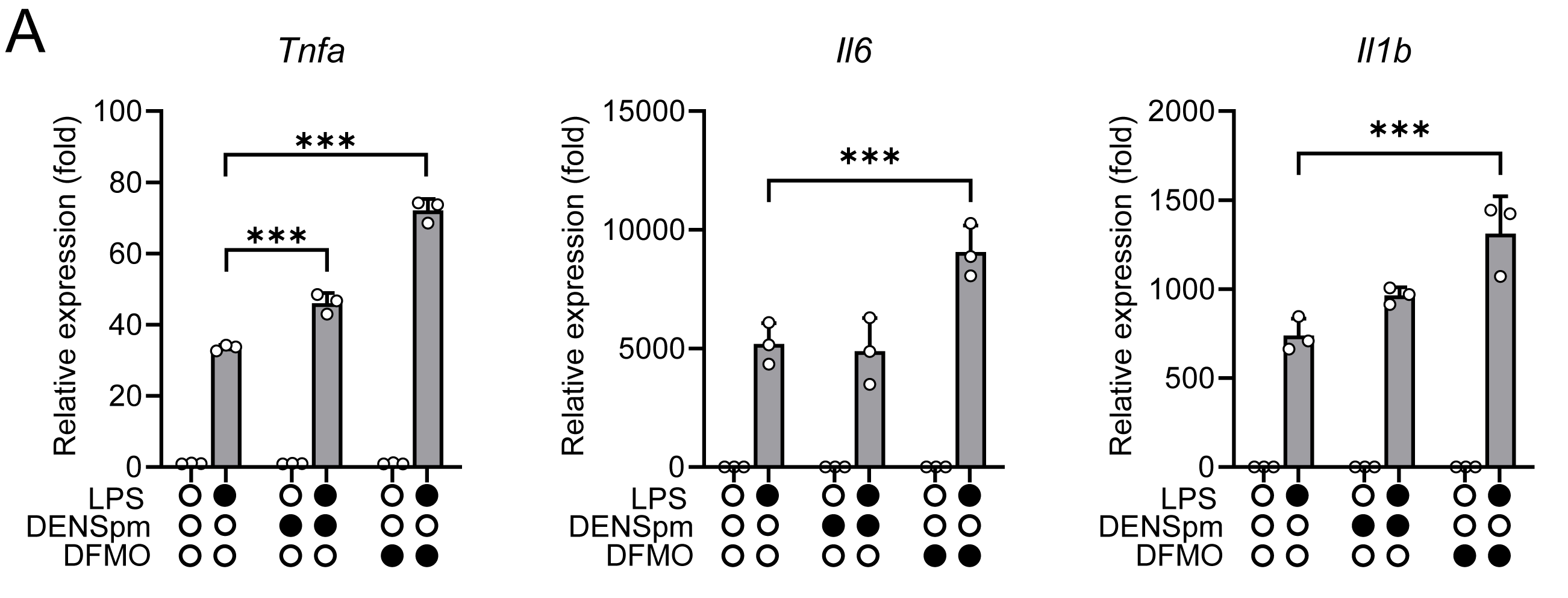

Supplement: Fig. S4 — Endogenous polyamines restrain LPS-induced innate responses in macrophages. [file mbio.00846-26-s0004.tif]

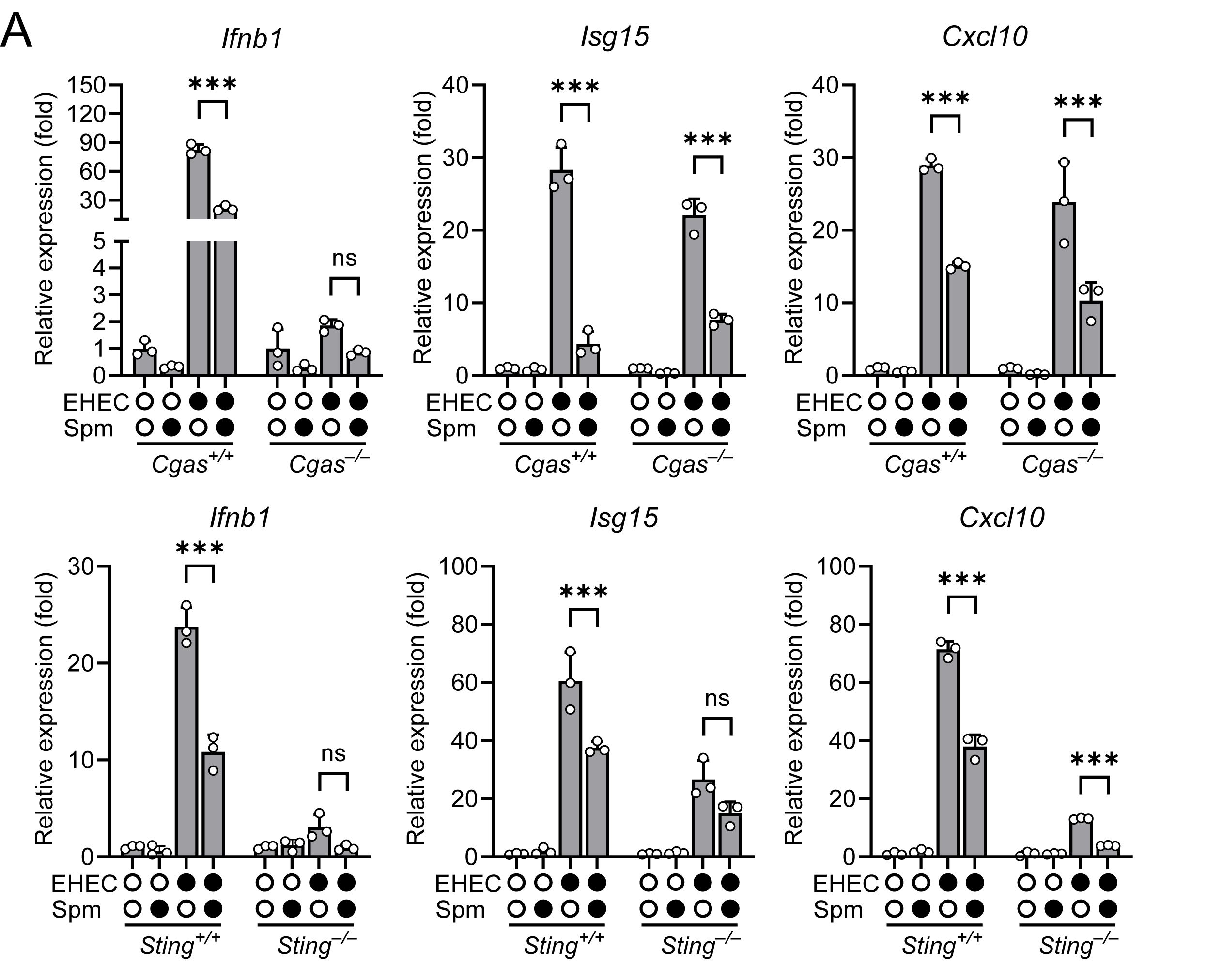

Supplement: Fig. S5 — Spermine-mediated immune suppression against EHEC infection is partially dependent on the cGAS-STING pathway. [file mbio.00846-26-s0005.tif]

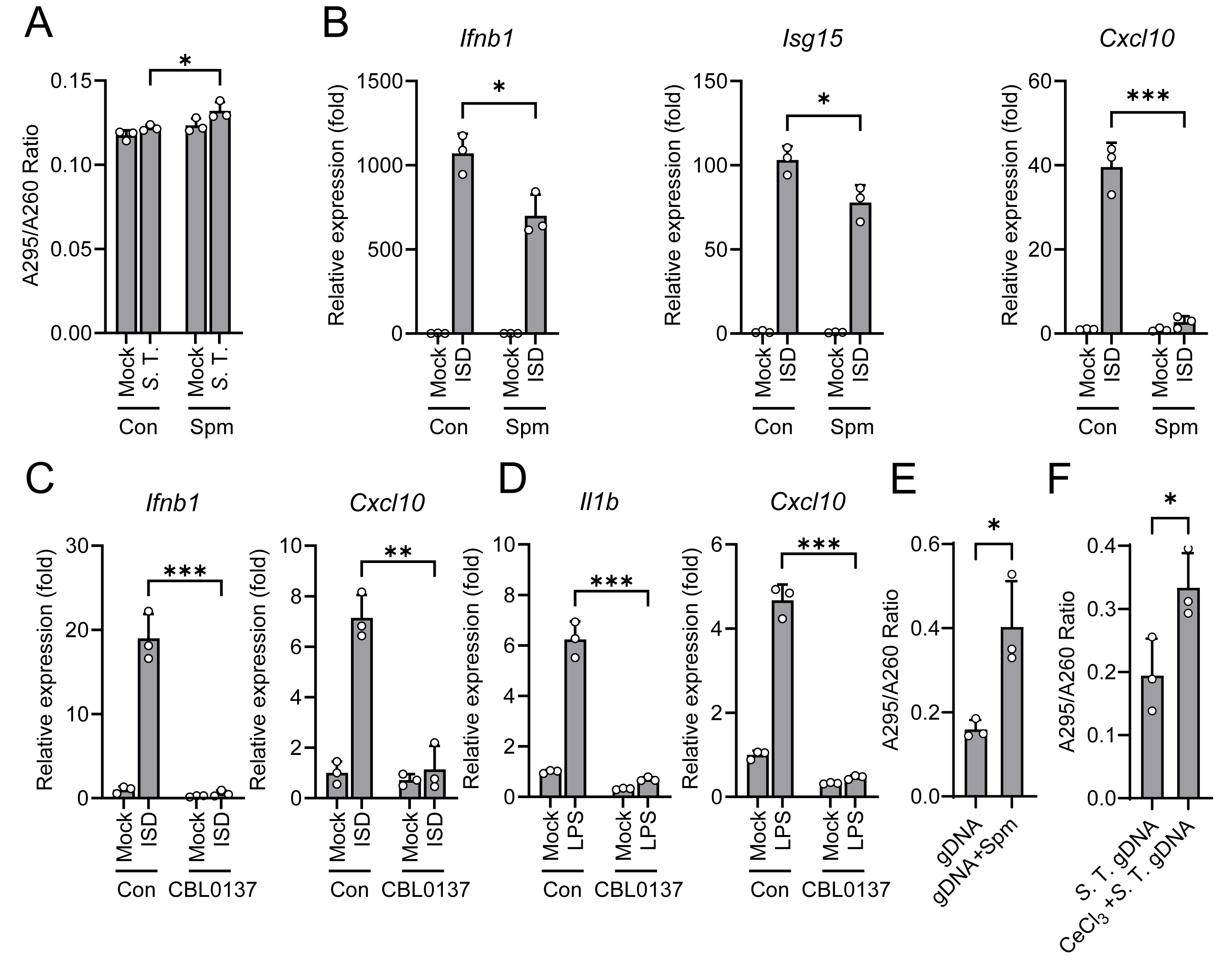

Supplement: Fig. S6 — Spermine and Z-DNA formation attenuate ISD-induced innate immune responses. [file mbio.00846-26-s0006.tif]

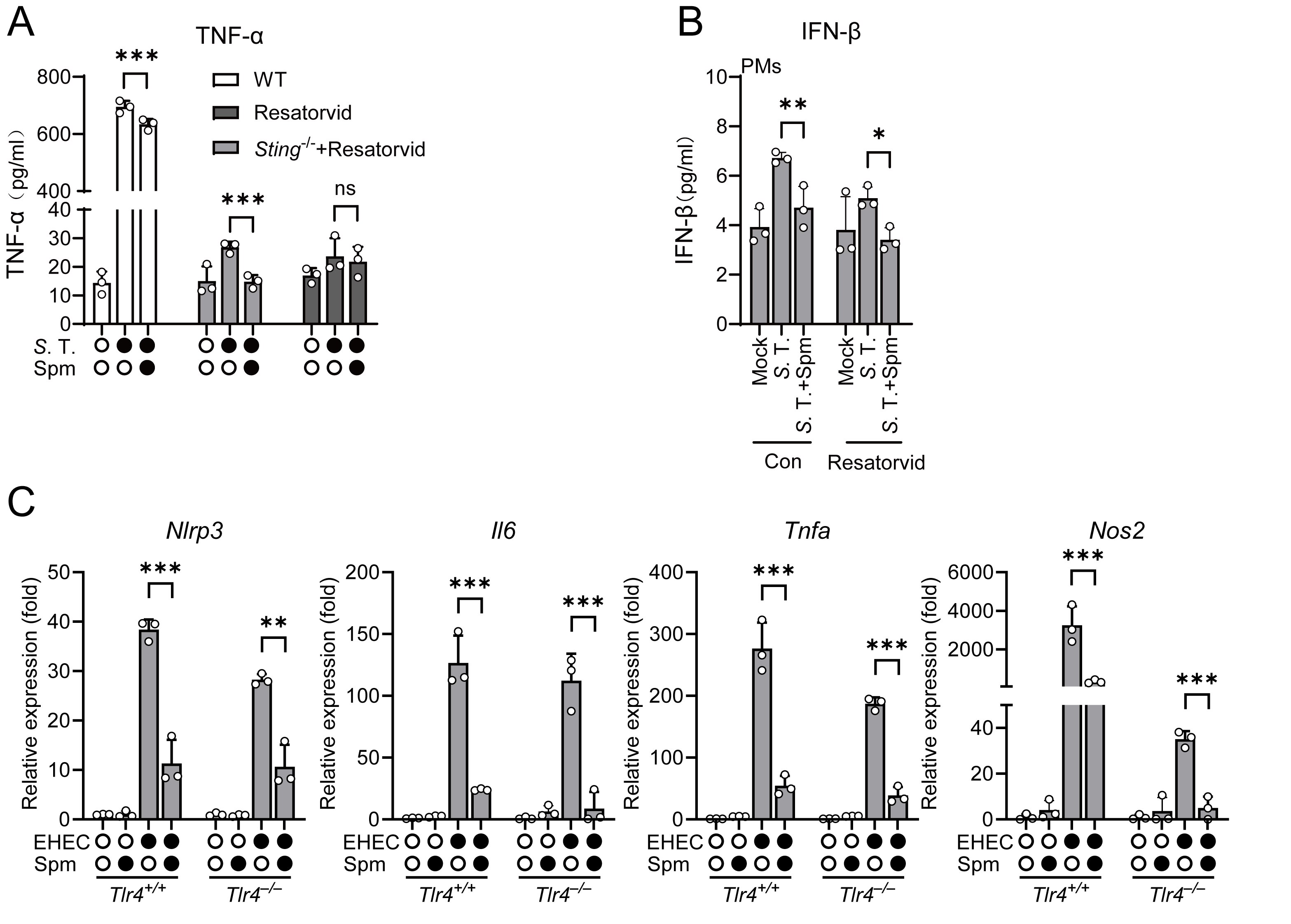

Supplement: Fig. S7 — Spermine inhibits EHEC-elicited innate immune response irrespective of TLR4 status. [file mbio.00846-26-s0007.tif]

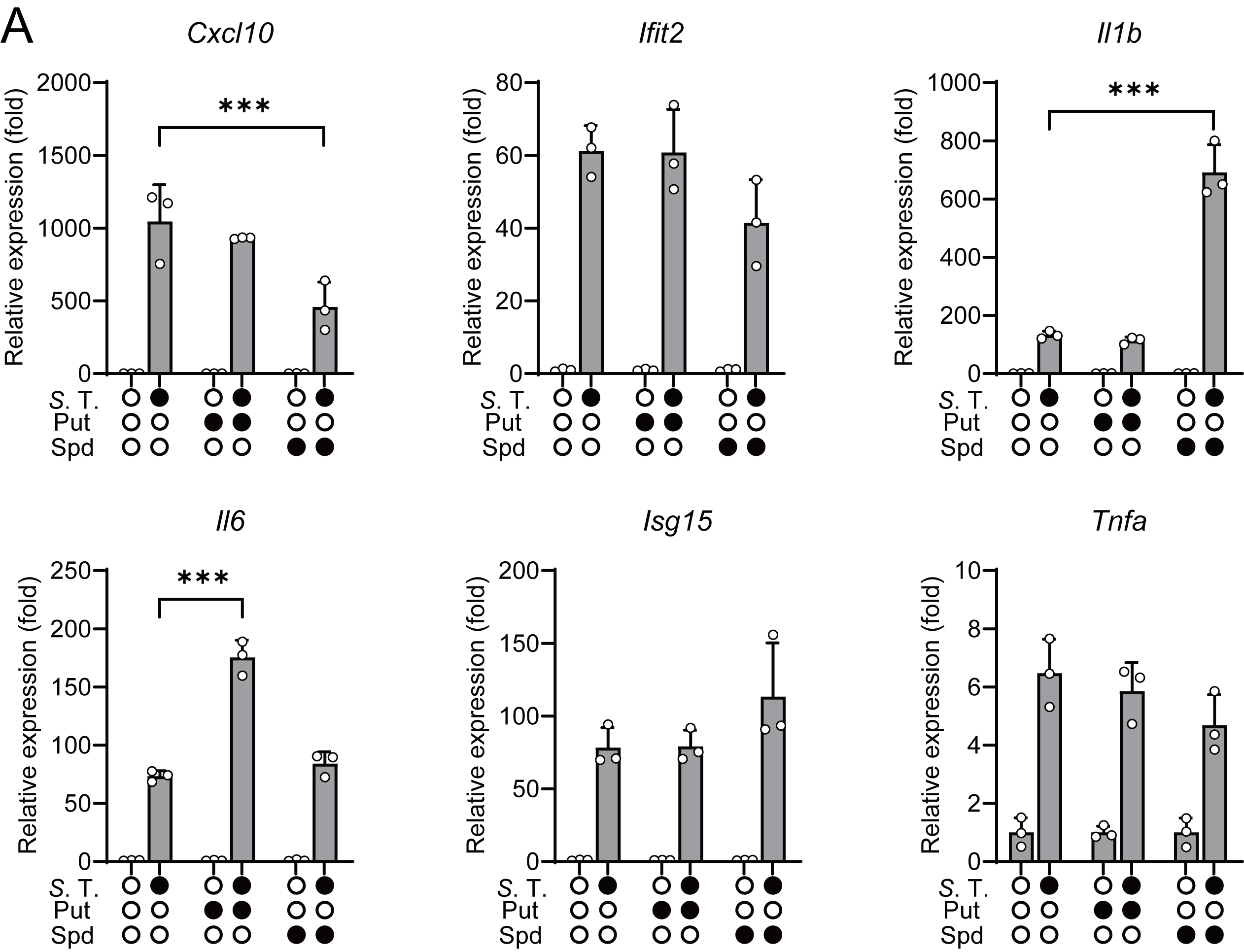

Supplement: Fig. S8 — Effects of putrescine and spermidine on S. Typhimurium-induced gene expression in macrophages. [file mbio.00846-26-s0008.tif]

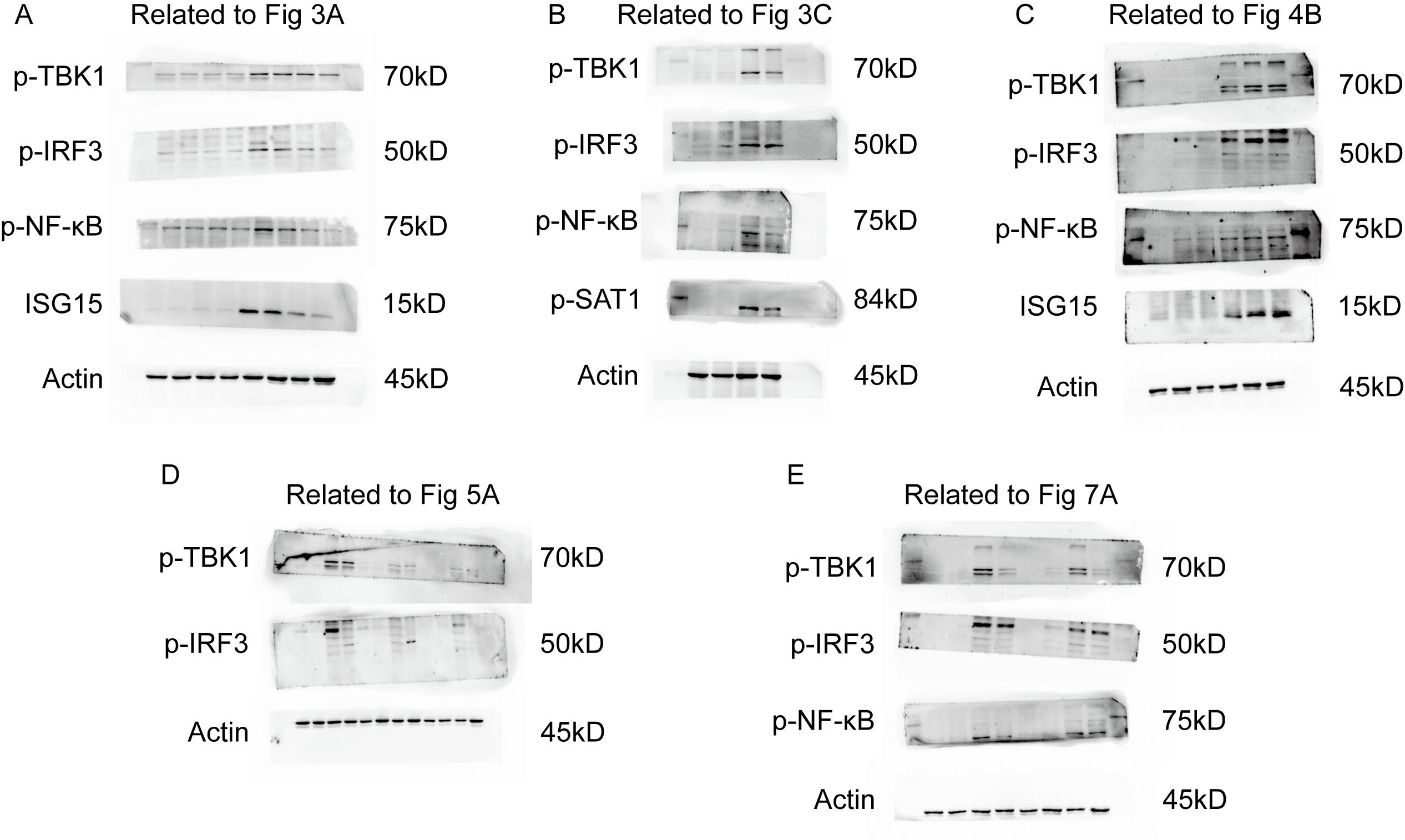

Supplement: Fig. S9 — Uncropped versions of immunoblotting results. [file mbio.00846-26-s0009.tif]
