# Supplementary material for: Spermine suppresses Salmonella-induced macrophage innate immune responses via inhibition of the cGAS-STING and TLR4 pathways
Source: mBio. 2026 May 29;17(7):e00846-26. doi: 10.1128/mbio.00846-26 (PMC13343856; doi:10.1128/mbio.00846-26)
Supplement: Supplemental material — Legends for Fig. S1-S9; Tables S1 and S2. [file mbio.00846-26-s0010.docx]

SUPPORTING INFORMATION CAPTIONS

S1 Fig. LPS stimulation rewires macrophage polyamine metabolism.

(A) Heatmap of the expression profiles of polyamine metabolism genes in LPS (100ng/mL, 6 h) versus Mock. *Z* scores of the relative gene expression levels are displayed, with red representing higher and blue representing lower abundance. n = 3.

Figure S2 spermine does directly not affect bacterial growth.

(A) Standard solutions of spermine at indicated concentrations (0–2.5 mM) were analyzed by HPLC. The standard curve was constructed by plotting the peak area against the corresponding concentration (mM).

(B) Growth curve of *S*. Typhimurium in LB medium with or without 100 μM spermine.

Figure S3 Spermine blunts pro-inflammatory response in macrophages.

(A) The mRNA expression levels of the indicated genes in PMs pretreatment with 100 μM spermine for 2 h and transfected with LPS (100ng/mL, 6 h), relative to Mock- transfected control. n = 3.

(B) The mRNA expression levels of the indicated genes in PMs pretreatment with 100 μM spermine for 2 h and infected with EHEC (MOI =10, 6 h), relative to Mock-infected control. n = 3.

(C) The mRNA expression levels of the indicated genes in RAW264.7 cells pretreatment with 100 μM spermine for 2 h and infected with *S*. Typhimurium (MOI =100, 6 h), relative to Mock-infected control. n = 3.

(D) The mRNA expression levels of the indicated genes in BMDMs pretreatment with 100 μM spermine for 2 h and infected with *S*. Typhimurium (MOI =10, 6 h), relative to Mock-infected control. n = 3.

(A)-(D), Student’s two-tailed t test.

Error bars represent ± SEM. *P < 0.05; **P < 0.01; ***P < 0.001.

Figure S4 Endogenous polyamines restrain LPS-induced innate responses in macrophage

(A) The mRNA expression levels of the indicated genes in PMs pretreatment with 10 μM DENSpm for 24 h or 500 μM DFMO for 24 h and transfected with LPS (100ng/mL, 6 h), relative to Mock- transfected control. n = 3.

(A), Student’s two-tailed t test.

Error bars represent ± SEM. *P < 0.05; **P < 0.01; ***P < 0.001.

Figure S5 Spermine-mediated immune suppression against EHEC infection is partially dependent on the cGAS-STING pathway

(A) The mRNA expression levels of the indicated genes in *Cgas*^+/+^ or *Cgas*^−/−^ and *Sting*^+/+^ or *Sting*^−/−^ mouse PMs pretreatment with 100 μM spermine for 2 h and infected with EHEC (MOI =10, 6 h), relative to Mock-infected control. n = 3.

(A), Student’s two-tailed t test.

Error bars represent ± SEM. *P < 0.05; **P < 0.01; ***P < 0.001. ns, non-significant

Figure S6 Spermine and Z-DNA formation attenuates ISD indued innate immune responses.

(A) PMs infected with *S*. Typhimurium (MOI =10, 6 h) were lysed and total DNA was extracted. The absorbance values were measured at 260 and 295 nm, and the A295/260 ratio was calculated.

(B) The mRNA expression levels of the indicated genes in PMs pretreatment with 100 μM spermine for 2 h and transfected with ISD (1 μg/mL, 6 h), relative to Mock- transfected control. n = 3.

(C) The mRNA expression levels of the indicated genes in PMs pretreatment with 5 μM CBL0137 for 2 h and transfected with ISD (1 μg/mL, 6 h), relative to Mock- transfected control. n = 3.

(E) Genomic DNA (gDNA) was incubated with 10 μM spermine, followed by buffer exchange. The absorbance values were measured at 260 and 295 nm, and the A295/260 ratio was calculated. n = 3.

(F) *S*. Typhimurium were cultured in LB with or without CeCl_3_ (500 μM) until the stationary phase and extracted genomic DNA. The absorbance values were measured at 260 and 295 nm, and the A295/260 ratio was calculated. n = 3.

(A) to (F), Student’s two-tailed t test.

Error bars represent ± SEM. *P < 0.05; **P < 0.01; ***P < 0.001.

Figure S7 Spermine inhibits EHEC-elicited innate immune response irrespective of TLR4 status.

(A) WT PMs and *Sting^−/−^* mouse PMs were first treated with 100nM resatorvid for 12 h，and then treated with 100 μM spermine for 2 h. Enzyme-linked immunosorbent assay (ELISA) analysis of TNF-α production in WT PMs and *Sting^−/−^* mouse PMs infected with *S*. Typhimurium (MOI =10, 6 h). n = 3.

(B) WT PMs were first treated with 100nM resatorvid for 12 h，and then treated with 100 μM spermine for 2 h. Enzyme-linked immunosorbent assay (ELISA) analysis of IFN-β production in WT PMs mouse PMs infected with *S*. Typhimurium (MOI =10, 6 h). n = 3.

(C) The mRNA expression levels of the indicated genes in *Tlr4*^+/+^ or *Tlr4*^−/−^ mouse PMs pretreatment with 100 μM spermine for 2 h and infected with EHEC (MOI =10, 6 h), relative to Mock-infected control. n = 3.

(A) to (C), Student’s two-tailed t test.

Error bars represent ± SEM. **P < 0.01; ***P < 0.001.

Figure S8. Effects of putrescine and spermidine on *S.* Typhimurium-induced gene expression in macrophages.

(A) The mRNA expression levels of the indicated genes in PMs pretreatment with 100 μM spermidine or 100 μM putrescine for 2 h and infected with *S*. Typhimurium (MOI =10, 6 h), relative to Mock- transfected control. n = 3.

(A), Student’s two-tailed t test.

Error bars represent ± SEM. *P < 0.05; **P < 0.01; ***P < 0.001.

Figure S9. Uncropped versions of immunoblotting results.

**Supplementary Table 1. Key resources used in this study.**

**Supplementary Table 2. Primers used in this study.**

**Supplementary Table 1. Key resources used in this study.**

| REAGENTS or RESOURCES | SOURCE | IDENTIFIER |
| --- | --- | --- |
| Chemicals, peptides, and recombinant proteins | | |
| Spermine | Sigma | #55513 |
| DFMO | MedChemExpress | #HY-B0744 |
| DENSpm | Aladdin | #N287060 |
| CBL0137 | Selleck | Cat#S0507 |
| Chloroquine | MedChemExpress | #HY-17589A |
| Ethidium bromide | MedChemExpress | #HY-D0021 |
| Lipopolysaccharide (*E. coli* O111:B4, LPS) | Beyotime | #S1732 |
| Sodium cacodylate trihydrate | Aladdin | #S464785 |
| Benzoyl chloride | Aladdin | #[B104565](https://www.aladdin-e.com/zh_cn/b104565.html) |
| EZ Cell Transfection Reagent | Life-iLab | #AC04L091 |
| Cerium Chloride | Aladdin | #C104760 |
| Resatorvid | MedChemExpress | #HY-11109 |
| Dimethyl sulfoxide (DMSO) | MedChemExpress | #HY-Y0320 |
| TRIzol Reagent | Sigma-Aldrich | #T9424 |
| Recombinant Murine M-CSF | Beyotime | #P6015 |
| Paraformaldehyde Fix Solution | Beyotime | #P0099 |
| Triton X-100 | Beyotime | #ST1723 |
| Immunostaining Blocking Buffer containing Saponin | Beyotime | #P0104 |
| Antifade Mounting Medium with DAPI | Beyotime | #P0131 |
| Medium and solution | | |
| Phosphate-Buffered Saline (PBS) | Beyotime | #C0221A |
| Penicillin-Streptomycin Solution (100×) | Beyotime | #C0222 |
| Dulbecco's Modified Eagle Medium (DMEM) High glucose (4500 mg/L) | GIBCO | #11965-084 |
| Opti-MEM I Reduced Serum Media | GIBCO | #11058-021 |
| RPMI 1640 | GIBCO | #11875-093 |
| Fetal Bovine Serum, FBS | Byotime | #C0234 |
| 100mM Sodium pyruvate Solution | Sigma-Aldrich | #S8636; CAS: 113-24-6 |
| 1M HEPES Solution | Sigma-Aldrich | #H0887; CAS: 7365-45-9 |
| MEM Non-Essential Amino Acids (100×) | GIBCO | #11140-050 |
| 2-Mercaptoethanol | Sigma-Aldrich | #M6250; CAS: 60-24-2 |
| QuickBlock™ Blocking Buffer | Beyotime | #P0252 |
| QuickBlock™ Primary Antibody Dilution Buffer for Western Blot | Beyotime | #P0256 |
| RIPA Lysis Buffer | Beyotime | #P0013B |
| Bacterial strains | | |
| *Salmonella* Typhimurium SL1344 | Our laboratory | N/A |
| *Escherichia coli* EDL933 | Our laboratory | N/A |
| Antibodies | | |
| Mouse monoclonal anti-β-actin (clone C4) | Santa Cruz Biotechnology | #sc-47778, RRID: AB_2714189 |
| Mouse monoclonal anti-ISG15 (F-9) | Santa Cruz Biotechnology | #sc-166755, RRID: AB_2126308 |
| Rabbit monoclonal anti-Phospho-TBK1/NAK (Ser172) (clone D52C2) | Cell Signaling Technology | #5483, RRID: AB_10693472 |
| Phospho-Stat1 (Tyr701) (58D6) Rabbit Monoclonal Antibody | Cell Signaling Technology | #9167; RRID: AB_561284 |
| Phospho-NF-κB p65 (Ser468) Recombinant monoclonal antibody | [proteintech](mailto:proteintech-cn@ptglab.com) | #82335-1-RR |
| Phospho-IRF3 (Ser396) Polyclonal antibody | [proteintech](mailto:proteintech-cn@ptglab.com) | #29528-1-AP |
| Anti-Z-DNA/Z-RNA [Z22] | Absolute Antibody | #Ab00783-23.0 |
| Goat anti-Rabbit IgG (H+L) secondary antibody | ZSGB-BIO | #ZF-0316 |
| Experimental Models: Organisms/Strains | | |
| Mouse: C57BL/6 | Beijing Vital River Laboratory Animal Technology | N/A |
| Mouse: STING^−/−^ C57BL/6 | Dr. Zhengfan Jiang | N/A |
| Mouse: cGAS^−/−^ C57BL/6 | Dr. Zhengfan Jiang | N/A |
| Mouse: TLR4^−/−^ C57BL/10ScNJGpt | GemPharmatech, Jiangsu, China | N/A |
| Experimental Models: Cell lines | | |
| Mouse: Raw264.7 cells | ATCC | #TIB-71 |
| Commercial Assays | | |
| RNAeasy™ Animal RNA Isolation Kit with Spin Column | Beyotime | #R0027 |
| RNAprep Pure Tissue Kit | TIANGEN | #DP431 |
| Reverse Transcription kit | Transgene | #AH311-02 |
| SYBR FAST qPCR Kit | KAPA Biosystem | #KK4601 |
| Mouse Interferon β, IFN-β/IFNB ELISA Kit | CUSABIO | #CSB-E04945m |
| Mouse Polyamine ELISA Kit | Yfxbio Biotech. Co. Ltd. | #YFXEM00089 |
| Protein Endotoxin Removal Kit | Beyotime | #C0268S |
| dsDNA HS Assay Kit | Yeasen | #12640ES60 |
| TIANamp Bacteria DNA Kit | TIANGEN | #DP302 |
| Oligonucleotides | | |
| Primers for qRT-PCR see Tables S2 | This paper | N/A |
| Other | | |
| GraphPad Prism 10.01 | GraphPad | https://www.graphpad.com/ |

**Supplementary Table 2. Primers used in this study.**

| **Primers** | **5’-3’ sequence** | **Function** |
| --- | --- | --- |
| *ISD-F* | TACAGATCTACTAGTGATCTATGACTGATCTGTACATGATCTACA | To generate ISD |
| *ISD-R* | TGTAGATCATGTACAGATCAGTCATAGATCACTAGTAGATCTGTA |  |
| *Actb-F* | CATTGCTGACAGGATGCAGAAGG | qRT-PCR primers for genes of *Mus musculus* |
| *Actb-R* | TGCTGGAAGGTGGACAGTGAGG |  |
| *Sat1-F* | AAGTGTCGCTGCAGCAGTAT |  |
| *Sat1-R* | AGCCTCCATCCCTCTTCACT |  |
| *Sms-F* | TTGGCAGAGAGTGACTTGGC |  |
| *Sms-R* | CACACAATATGCCTCCATCGC |  |
| *Odc1-F* | TGCCAGTAACGGAGTCCAGA |  |
| *Odc1-R* | ATGTGCTCTGGCGACTTTCA |  |
| *Paox-F* | GGTTTATTGCTGGGCTGGAGT |  |
| *Paox -R* | GCTGTAGGAACCTCGGGTG |  |
| *Il1b-F* | TGGACCTTCCAGGATGAGGACA |  |
| *Il1b-R* | GTTCATCTCGGAGCCTGTAGTG |  |
| *Nlrp3 -F* | TCACAACTCGCCCAAGGAGGAA |  |
| *Nlrp3 R* | AAGAGACCACGGCAGAAGCTAG |  |
| *Tnfa-F* | GGTGCCTATGTCTCAGCCTCTT |  |
| *Tnfa-R* | GCCATAGAACTGATGAGAGGGAG |  |
| *Ifit1-F* | TACAGGCTGGAGTGTGCTGAGA |  |
| *Ifit1-R* | CTCCACTTTCAGAGCCTTCGCA |  |
| *Il6-F* | TACCACTTCACAAGTCGGAGGC |  |
| *Il6-R* | CTGCAAGTGCATCATCGTTGTTC |  |
| *Ifnb1-F* | GCCTTTGCCATCCAAGAGATGC |  |
| *Ifnb1-R* | ACACTGTCTGCTGGTGGAGTTC |  |
| *Isg15-F* | CATCCTGGTGAGGAACGAAAGG |  |
| *Isg15-R* | CTCAGCCAGAACTGGTCTTCGT |  |
| *Cxcl10-F* | ATCATCCCTGCGAGCCTATCCT |  |
| *Cxcl10-R* | GACCTTTTTTGGCTAAACGCTTTC |  |
| *Ifit2-F* | CGAACTACCGTCTGGATGACTG |  |
| *Ifit2-R* | CTTCAACCAGCGCCATTGCTTG |  |
